# The impact of cardiovascular diagnostics and treatments on fall risk in older adults: a scoping review and evidence map

**DOI:** 10.1007/s11357-023-00974-4

**Published:** 2023-10-21

**Authors:** Anouschka C. Pronk, Liping Wang, Eveline P. van Poelgeest, Mariska M. G. Leeflang, Joost G. Daams, Alfons G. Hoekstra, Nathalie van der Velde

**Affiliations:** 1 Department of Internal Medicine, Section of Geriatrics, Amsterdam University Medical Centers, Location University of Amsterdam, Meibergdreef 9, Amsterdam, The Netherlands; 2 Amsterdam Public Health Research Institute, Aging and Later Life, Amsterdam, The Netherlands; 3 Department of Epidemiology and Data Science Section of Methodology, Amsterdam University Medical Centres, Location University of Amsterdam, Meibergdreef 9, Amsterdam, The Netherlands; 4 Medical Library, University of Amsterdam, Amsterdam, The Netherlands; 5 Computational Science Lab, Informatics Institute, University of Amsterdam, Amsterdam, The Netherlands

**Keywords:** Cardiovascular, Intervention, Older adults, Fall prevention, Evidence map, Scoping review

## Abstract

**Background:**

We aimed to summarize the published evidence on the fall risk reducing potential of cardiovascular diagnostics and treatments in older adults.

**Methods:**

*Design:* scoping review and evidence map. *Data sources:* Medline and Embase. *Eligibility criteria:* all available published evidence; Key search concepts: “older adults,” “cardiovascular evaluation,” “cardiovascular intervention,” and “falls.” Studies reporting on fall risk reducing effect of the diagnostic/treatment were included in the evidence map. Studies that investigated cardiovascular diagnostics or treatments within the context of falls, but without reporting a fall-related outcome, were included in the scoping review for qualitative synthesis.

**Results:**

Two articles on cardiovascular diagnostics and eight articles on cardiovascular treatments were included in the evidence map. Six out of ten studies concerned pacemaker intervention of which one meta-analyses that included randomized controlled trials with contradictory results. A combined cardiovascular assessment/evaluation (one study) and pharmacotherapy in orthostatic hypotension (one study) showed fall reducing potential. The scoping review contained 40 articles on cardiovascular diagnostics and one on cardiovascular treatments. It provides an extensive overview of several diagnostics (e.g., orthostatic blood pressure measurements, heart rhythm assessment) useful in fall prevention. Also, diagnostics were identified, that could potentially provide added value in fall prevention (e.g., blood pressure variability and head turning).

**Conclusion:**

Although the majority of studies showed a reduction in falls after the intervention, the total amount of evidence regarding the effect of cardiovascular diagnostics/treatments on falls is small. Our findings can be used to optimize fall prevention strategies and develop an evidence-based fall prevention care pathway. Adhering to the World guidelines on fall prevention recommendations, it is crucial to undertake a standardized assessment of cardiovascular risk factors, followed by supplementary testing and corresponding interventions, as effective components of fall prevention strategies. In addition, accompanying diagnostics such as blood pressure variability and head turning can be of added value.

**Supplementary Information:**

The online version contains supplementary material available at 10.1007/s11357-023-00974-4.

## Introduction

Among older adults worldwide, falls are a major health threat and a leading cause of morbidity and mortality [1]. Each year, approximately 30% of people aged 65 or older experience at least one fall, with half of those aged 80 and above suffering from one or more falls [2, 3]. Severe injuries, such as hip fractures or head trauma, occur in around 10% of fallers [4, 5], resulting in an increased burden on both medical resources and finances [1, 6, 7]. Given the projected increase in fall-related injuries in the coming years, it is expected that healthcare consumption related to falls will further rise [1].

Cardiovascular conditions such as orthostatic hypotension, vasovagal syndrome, carotid sinus hypersensitivity (CSH), and arrhythmias are consistently linked to fall risk in older people, particularly in unexplained and recurrent falls [2, 8, 9]. Data from patient surveys and medical record-based analyses identify falls or risk of falling in 40 to 60% of adults with cardiovascular disease [10]. The differentiation between syncope and falls is often challenging: eyewitnesses are often lacking, and older individuals frequently suffer from retrograde amnesia after a syncopal event and tend to report a fall [11–13]. Therefore, both the 2018 European Society of Cardiology (ESC) guideline on syncope [14] and the 2022 World Falls Guidelines (WFG) [2] recommend the same cardiovascular work-up for unexplained falls as for syncope. Nevertheless, in current clinical practice, this is not routinely performed, and considerable practice variation exists between clinics and clinicians. In general, cardiovascular fall risk assessment includes detailed history taking, physical examination, testing for orthostatic hypotension (OH), and a 12-lead electrocardiogram (ECG). On indication, and depending on available resources, echocardiography, carotid sinus massage (CSM), tilt table testing and/or other cardiovascular testing is performed [2]. A recent systematic review and (network) meta-analysis [15] on the fall risk reducing effects of various interventions showed a fall risk reducing effect of cardiovascular interventions (e.g., including vital signs, ECG, loop recorder). However, an overview focusing solely on the fall risk reducing effects of cardiovascular assessments and treatments is currently lacking.

In this scoping review and evidence map, we will update and extent our previous work [16], by summarizing the published evidence on the fall risk reducing potential of cardiovascular diagnostics and treatments in older adults. Evidence mapping (EM) is an evolving methodology, suitable for summarizing published evidence and research activity in broad topic areas, and for identification and visualization of research gaps to guide evidence-based decision-making [17–19].

## Methods

A protocol paper for this scoping review and evidence map was published in advance [20]. The scoping review was conducted and reported in accordance with the Preferred Reporting Items for Systematic reviews and Meta-analyses extension for Scoping Reviews (PRISMA-ScR) checklist [21, 22] (Supplementary Appendix A). The evidence map was based on the guidance for producing a Campbell evidence and gap map [23].

### Search strategy

The search strategy (Supplementary Appendix B) was developed by the project team under the guidance of an experienced medical librarian (JD). The search strategy included three concepts: (1) older adults; (2) cardiovascular diagnostics/interventions; and (3) falls*.* Potentially eligible articles were systematically searched in MEDLINE and EMBASE from inception to June 30, 2022. Reference lists from included review papers were checked for additional potentially eligible studies.

### Eligibility criteria

A detailed description of the eligibility criteria can be found in the published protocol paper [20]. In addition, for the evidence map, studies that investigated cardiovascular diagnostics/treatment with a fall-related outcome were included. Studies that investigated cardiovascular diagnostics or treatments within the context of falls (e.g., diagnostics used in patients presenting with unexplained falls), but without reporting a fall-related outcome, were included in the scoping review.

### Study types

All available published evidence (e.g., observational studies, (non-) randomized controlled trials) in all settings (community dwelling, hospital, and long-term care facility) were considered. In addition, systematic reviews and meta-analyses were considered. In case a systematic review and/or meta-analysis was included in a certain category of cardiovascular diagnostics or treatments, only studies were considered that were published after publication of the systematic review and/or meta-analysis, or in case a study had additional value on the topic.

### Study selection and data extraction

Title/abstract screening was conducted by two reviewers (LW and AP). First, three screening rounds (6% of abstracts) were performed by the two reviewers independently, optimizing the inclusion and exclusion criteria, and calibrating inter-individual screening accuracy. After reaching a near-complete (>99%) agreement, the remainder of references were divided between the two reviewers, and single-screened. Full-text review was performed by the two reviewers independently to assess all full-text articles for eligibility. In case of disagreement and uncertainties in both screening phases, a third reviewer (EvP) was consulted. The two reviewers independently extracted data using predefined standardized data collection forms. The extracted information included relevant study characteristics and results (e.g., study design, setting, age, intervention type, and (fall-related) outcomes. In case the required data was missing, incomplete, or unclear, inquiries were sent to the corresponding author.

### Data synthesis and mapping

The extracted data were organized and summarized in separate descriptive tables according to the investigated cardiovascular diagnostic or treatment. In the evidence (and gap) map, the impact of the interventions on reducing fall risk is visually displayed. This EM also shows the related cardiovascular diagnostic or treatment approaches, study designs, sample sizes of the included studies. The studies included in the scoping review are summarized in a qualitative synthesis.

### Critical appraisal of studies included in evidence map

Two independent reviewers (LW and AP) assessed the quality of studies included in the EM. The Cochrane Checklist assessing Risk of bias (ROB 2) [24] was used for randomized controlled trials (RCTs), and the Risk of bias in non-randomized studies of interventions (ROBINS-I) tool [25] was used for non-randomized intervention studies. The Joanna Briggs Institute (JBI) critical appraisal tool [26] was used for observational studies. The A MeaSurement Tool to Assess systematic Reviews (AMSTAR 2) checklist [27] was used for critical appraisal of included systematic reviews. Disagreements between the reviewers were discussed with the third reviewer (EvP) and solved with consensus. Details of the critical appraisal are described in Supplementary Appendix C.

## Results

### Search result and study selection

**Fig. 1:**
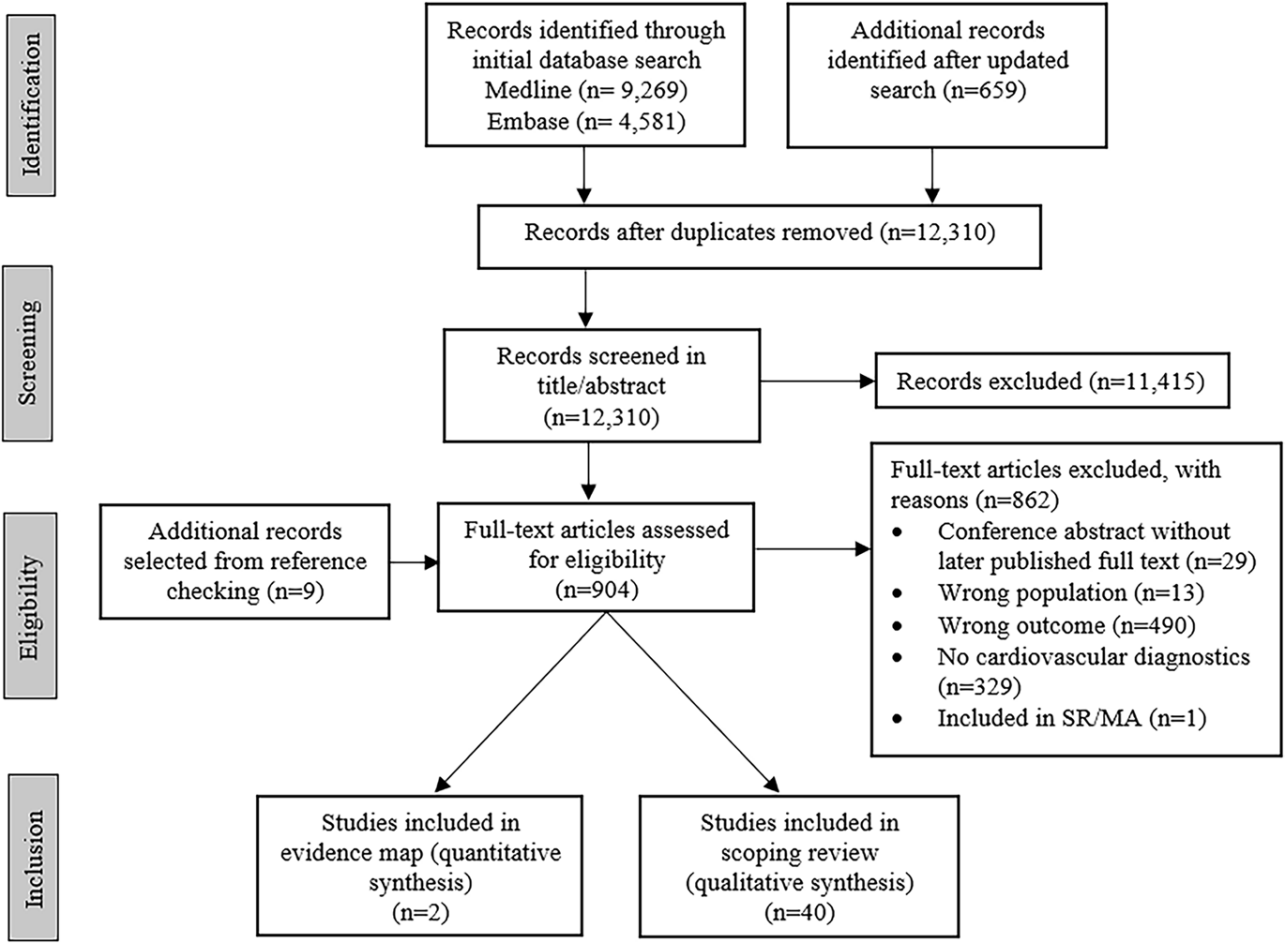
Flow diagram of the study selection process for fall-preventive cardiovascular diagnostics. SR/MA: systematic review/meta-analysis

**Fig. 2:**
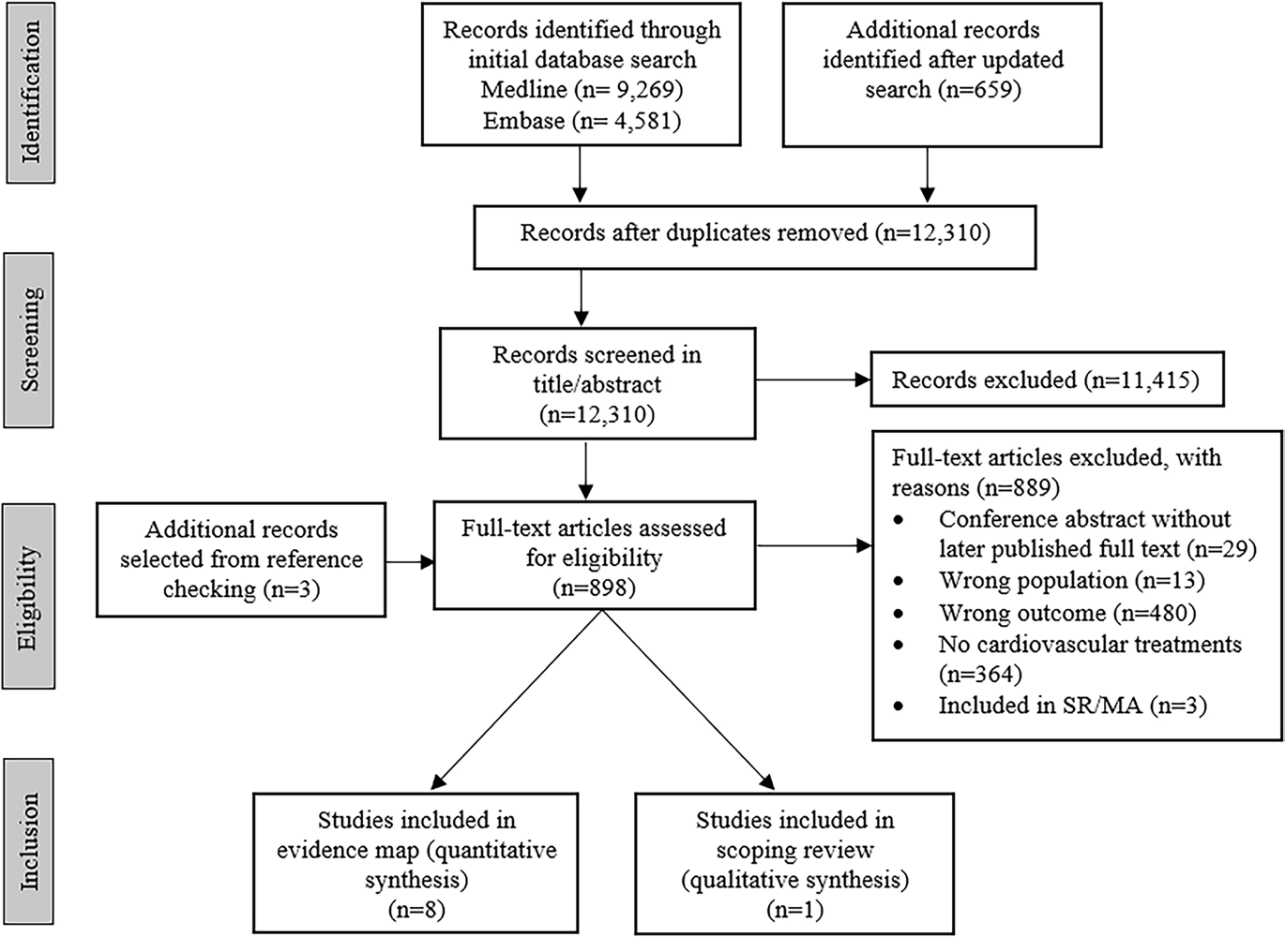
Flow diagram of the study selection process for fall-preventive cardiovascular treatments. SR/MA: systematic review/meta-analysis

**Fig. 3:**
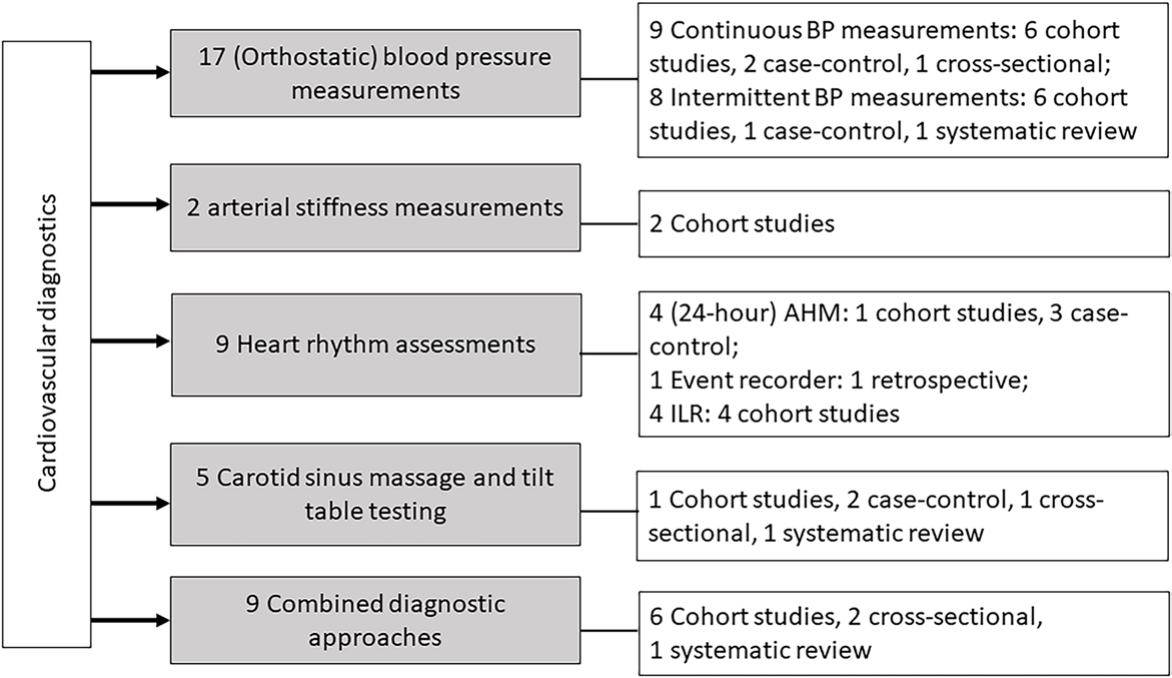
Summary of included papers on cardiovascular diagnostics for fall prevention. AHM: ambulatory Holter monitoring, BP: blood pressure, ILR: implantable loop recorder

**Fig. 4:**
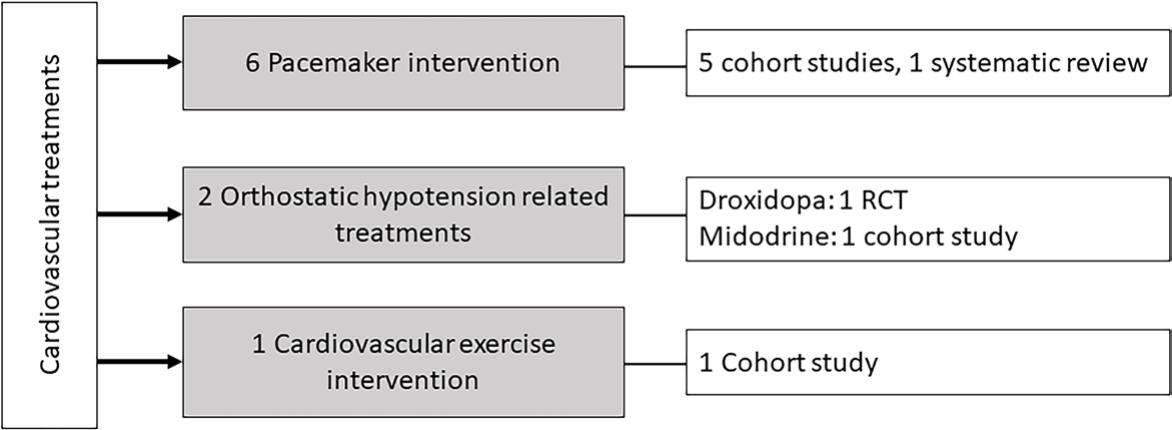
Summary of included papers on cardiovascular treatments for fall prevention. RCT: randomized controlled trial

Our search resulted in 12,310 unique records, which underwent screening and assessment for eligibility (see Fig. 1 and Fig. 2). Of these, 10 papers were included in the EM, while 41 papers were included in the scoping review. Of the included papers, 42 (82.4%) focused on cardiovascular diagnostics, 9 (17.6%) on cardiovascular treatments. The distribution of the included studies across the different categories is shown in Figs. 1, 2, 3, 4.

#### Evidence map

##### Search results

**Fig. 5:**
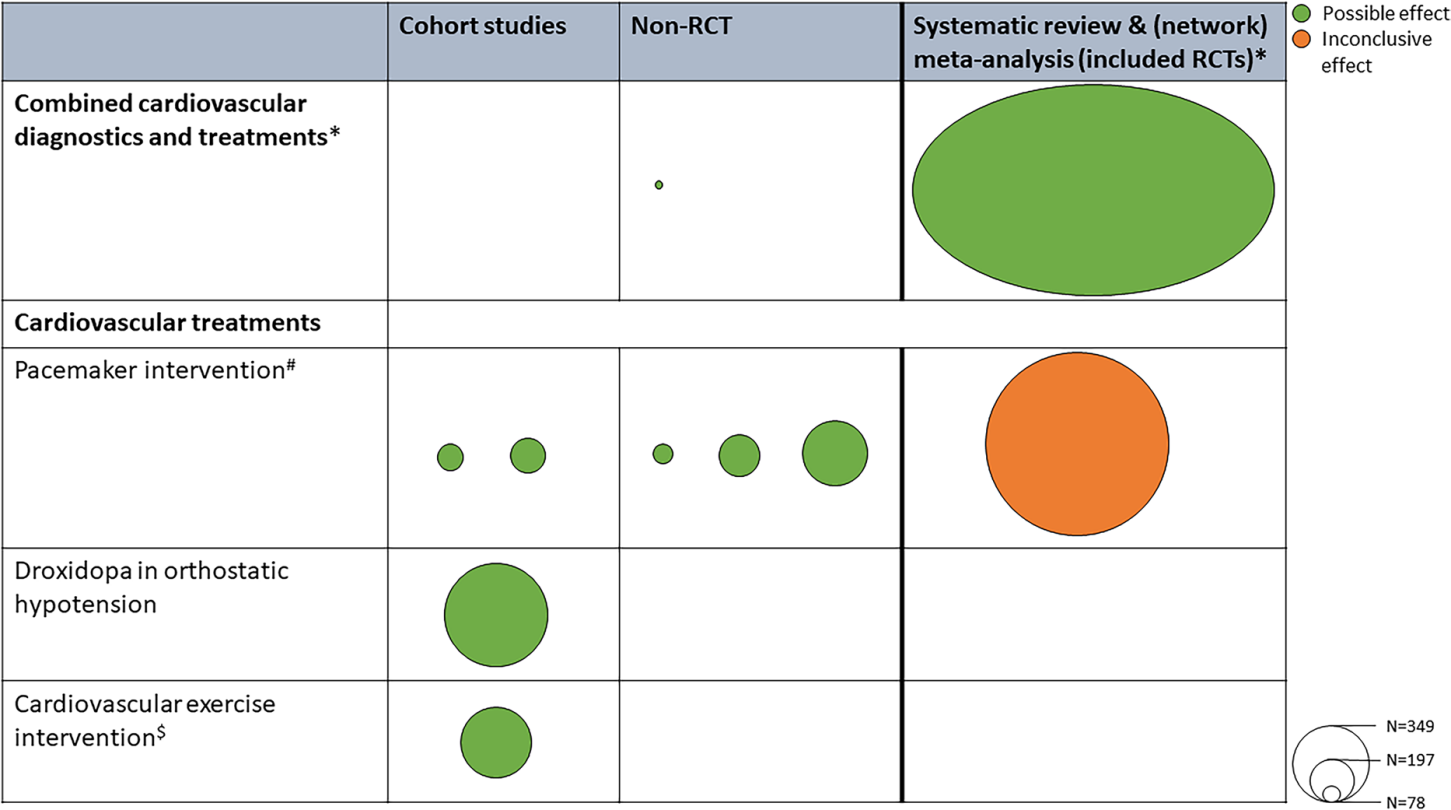
Evidence map for the effect of cardiovascular diagnostics and treatments on fall risk in older adults. Legend: Each circle represents a single study. The circle size represents the study sample size (N). A green circle indicates evidence of a possible effect, an orange circle indicates an inconclusive effect. * This circle is not proportional in size compared to the other included studies, due to large sample size (*N = *1592 participants). ^#^The systematic review & meta-analysis included three RCTs that reported contradictory results. ^$^The included study reported on improvement in risk of falls using the Morse Fall Scale. RCT: randomized controlled trial

Ten studies were included in the EM (eight original studies [28–35] and two systematic reviews and (network) meta-analyses [15, 36]); two studies for cardiovascular diagnostics and eight for cardiovascular treatments). The EM is shown in Fig. 5.

### Characteristics of included studies

Details of the included studies on study characteristics and results and critical appraisal are summarized in Supplementary Appendix D (Tables 1, 2, 3, 4) and Appendix C. Two studies were meta-analysis, one study was an RCT, and seven were cohort studies of which three were prospective and four were retrospective. Of the original studies, two studies were conducted at a falls clinic, one at a geriatric department, three at a cardiology department, one at Parkinson’s clinics, and one at a rehabilitation unit. Data on falls were collected prospectively in four, and retrospectively in four studies. The included systematic reviews and/or meta-analyses had low risk of bias according to the AMSTAR 2 checklist. The included non-randomized trials and observational studies had moderate or high risk of bias on at least one domain according to the ROBINS-I checklist and JBI critical appraisal tool.

### Combined assessments and treatments

We included one small (*n *= 15) prospective cohort study [33] in older falls clinic patients evaluating the efficacy of a comprehensive cardiovascular assessment and intervention, consisting of structured history taking, ECG, echocardiography, tilt table testing with continuous BP monitoring, followed by a multidisciplinary evaluation with treatment advice. In almost half of the fallers, this approach resulted in identification of a modifiable cardiovascular abnormality contributing to fall risk. All patients fell before the assessment and intervention, while 33% of patients experienced a fall after the evaluation and intervention. In addition, we included a systematic review and network meta-analysis [15], which reported on the fall risk reducing effect of different interventions used in fall prevention. Interventions addressing abnormalities found in cardiovascular components (called “basic fall risk assessment” including vital signs, ECG, loop recorder, medication review) were significantly associated with a reduction in number of fallers and falls rate. “Basic fall risk assessment” as a single intervention was most strongly associated with reduction in number of fall-related fractures (RR 0.60, 95% CI 0.39–0.94). These results suggest that combined cardiovascular assessments/treatments have fall reducing potential.

### Pacemaker intervention

We included one systematic review and meta-analyses [36] and five original studies [28–32] on the fall-risk reducing effect of pacemaker implantation in older adults. Indications for pacemaker implantation were CSH in six studies, and sinus node dysfunction (SND) in two studies.

In the meta-analysis [36], which included all existing RCTs evaluating the effect of pacemaker implantation in older patients with CSH and unexplained falls, cardiac pacing was not associated with reducing the risk of falling (RR 1.20, 95% CI 0.92–1.55), but did show a significant reduction in the rate of falls (rate ratio (RaR) 0.72, 95% CI 0.57–0.93). One (1/8) non-randomized study [30], evaluating the effect of pacemaker implantation in patients with CSH and recurrent falls, found that 81% of patients with CSH experienced falls in the year before implantation compared with 30% after implantation (*p *< 0.001). Two (2/8) studies [31, 32] retrospectively evaluated fall risk reduction of pacemaker implantation. In one study [31]*,* the number of falls prior and post dual chamber pacemaker implantation was compared. All patients (*n *= 7, unexplained falls as indication) experienced no more falls after pacemaker implantation. Another study [32] evaluated the effect of pacemaker implantation on symptoms in patients with carotid sinus syndrome (CSS). Number of falls reduced significantly post-implantation (21 pre-implant vs 8 post-implant; *p *= 0.043). Two (2/8) studies [28, 29] examined the effect of pacemaker implantation on fall risk in patients with SND. Both were non-randomized intervention studies assessing fall rates after at least 12 months of follow-up after pacemaker implantation. Both studies reported a significant decrease in fall rates, total number of falls, and fall-related injuries in patients with a pacemaker implanted.

### OH-related treatments

We included one systematic review and network meta-analysis [15] and one original study [35]. In the network meta-analysis by Dautzenberg et al. [15], OH management was one of the studied interventions. OH management as a single intervention was not associated with reducing fall risk, but OH treatment was one of the effective components (in different combinations) in reducing falls of multidomain interventions. The original study was a phase 3, RCT of droxidopa in 197 older (mean age 72 year) patients with Parkinson’s disease and symptomatic neurogenic OH [35]. Assessment of falls was originally included as a secondary endpoint, where they found less falls in the droxidopa group (droxidopa 308 falls compared to 908 falls in placebo group). Fall rates were 0.4 falls per patient week in the droxidopa group vs 1.05 falls per patient week in the placebo group (*p *= 0.014) and fall related injury was 16.7% in the droxidopa group vs 26.9% in placebo group.

### Cardiovascular exercise intervention

We included one study [34] on the effects (including fall risk) of a cardiac rehabilitation program (*n *= 135). Patients (mean age 80 years) after a transcatheter aortic valve implantation (TAVI) procedure were compared to patients after surgical aortic valve replacement (sAVR). Risk of falls was assessed (using Morse fall scale) at admission and discharge. Compared to sAVR, risk of falls was higher in patients after TAVI, both on admission (MFS: 36 ± 22 vs 25 ± 19; *p *= 0.002) and at discharge (MFS: 30 ± 20 vs 20 ± 12; *p *= 0.0001). Also, there was a significant improvement in risk of falls at discharge both in TAVI (MFS difference admission vs discharge: 5.8 ± 20; *p *= 0.02) and sAVR (MFS difference 5 ± 15; *p *= 0.005).

#### Scoping review

##### Search results

In total, 41 studies were included in the scoping review (39 original studies and two systematic reviews), 40 for cardiovascular diagnostics and one for cardiovascular treatment.

### Characteristics of included studies

Details of the 41 included studies on study characteristics and results are summarized in Supplementary Appendix D (Tables 1, 3, 5, 6, 7, 8). One study was a systematic review and meta-analyses, one a systematic review, 27 were cohort studies, four were cross-sectional studies, and eight were case control studies. Of the original studies, four studies were conducted at an emergency department (ED), one at a movement disorder clinic, four at a falls and syncope clinic, three at a geriatric department, 11 in the community, five at a geriatric outpatient clinic, one at a cardiology department, five at a long-term health care facility, one at an ED, geriatric outpatient clinic a primary care combined, two at a syncope unit and two at a hospital department. Data on falls were collected prospectively in seven and retrospectively in 32 studies.

#### Single diagnostic approaches

##### Tests for measuring (orthostatic) blood pressure

We included one systematic review and meta-analysis [37] and 16 original studies [38–53] on (orthostatic) blood pressure measurements in fallers. In the systematic review and meta-analysis by Mol et al. [37], the relevance of blood pressure measurement methodology in OH-related falls was demonstrated. The association between OH and falls was the strongest in the studies using continuous blood pressure measurements (OR 2.35, 95% CI 1.76–3.13) compared to intermittent blood pressure measurements (OR 1.66, 95% CI 1.43–1.93).

Continuous blood pressure measurements were used in six (6/16) studies [43–45, 47, 49, 51]. In three of these studies [44, 49, 51], blood pressure was measured continuously during active stand (AS) and the association between OH variants (e.g., initial OH, OH at 40 s) and falls was assessed. In all three studies, participants rested in supine position for 10 min and remained standing for 2 min. In two studies [44, 49], OH at 40 s (OH40) was an independent risk factor for future falls. In the first study, OH40 was associated with unexplained falls (RR 1.58, 95% CI 1.03–2.26) [44]. In the second study, OH40 was associated with unexplained (RR 1.57, 95% 1.16–2.13), recurrent (RR 1.35, 95% CI 1.08–1.68), and injurious falls (RR 1.44, 95% CI 1.16–1.78) [49]. In both studies, sustained OH was associated with unexplained falls. Initial OH was not associated with any fall related outcome [44]. In the third study [51], AS patterns were identified based on systolic blood pressure (SBP) deficits during AS. A composite of three AS-deficits ((1) difference in SBP ≥40mmHg at 10 s; (2) difference in SBP ≥20mmHg of supine levels at 40 s; (3) difference in SBP ≥20mmHg at any time between 50 and 120 s) was associated with fall history (OR 1.54, 95% CI 1.15–2.07). These results suggest that identifying OH variants, using continuous blood pressure measurements, may aid in estimating fall risk.

One study [47] showed that orthostatic BP drop rate (largest amplitude of the negative peak in the first derivative of BP) after standing (5 min supine rest and 3 min standing) was associated with number of falls in the previous year (*β* 0.30; 95% CI, 0.11–0.49), suggesting that BP drop rate during continuous blood pressure measurements could be a clinically relevant parameter linked to fall risk. In two studies [43, 45]*,* beat-to-beat blood pressure variability (BPV) in relation to falls was the focus of interest. BP was recorded during 10 min supine rest and 3 min standing. In one of these studies [43], new methods for calculating BPV were introduced (root mean square of real variability (RMSRV) and standard deviation of real variability (SDRV)) and found that these may be potential predictors for falls. The other study [45] found that reduced beat-to-beat BPV while standing was independently associated with increased risk of falls. Authors suggested that reduced beat-to-beat BPV may be a newly discovered fall risk factor.

Continuous blood pressure measurements during tilt table testing were performed in one (1/16) study [42]. This study sought for the finometrically measured time average best associated with OH-related falls. They found that the 5-s average performed best in relation to falls history (OR 2.54, 95% CI 1.37–4.71).

Intermittent (sphygmomanometer) blood pressure measurements during AS were used in four (4/16) studies [48, 50, 52, 53]. In one study [50], prevalence of OH was higher at the 30s measurement compared to the 3 min measurement. Both OH at 30 s (IRR 1.35, 95% CI 1.10–1.66) and at 3 min (IRR 1.48, 95% CI 1.04–2.02) after standing was associated with future fall risk. Authors concluded that the 30-s measurement was more reliable to detect fall risk. Another study [53] did not find an association between BP recovery (BP at 3 min minus BP at 1 minute after standing) and number of falls in the past year, suggesting that sphygmomanometer measurements have inadequate time resolution to record clinical relevant dynamics of orthostatic BP recovery. Another study [52], compared seated-to-standing measurements to supine-to-standing measurements. Supine-to-standing measurements appeared to better predict falls than seated-to-standing measurement (OH 2.1% (SE 0.5) in seated protocol vs 15% (SE 1.4) in supine protocol (*p *< 0.001)). The last study [48] examined the association between BP components and injurious falls. BP measurements were performed after five min supine rest and after one min standing. SBP <130mmHg, SBP >160mmHg, and high pulse pressure were associated with increased risk of injurious falls.

Intermittent (sphygmomanometer) blood pressure measurements at fixed times after a meal were performed to study postprandial hypotension (PPH), in three (3/16) studies [38, 39, 41]. In one study [38], nursing home residents with a history of syncope/falls had a greater maximum decrease of SBP compared to residents without a history of syncope/falls (21 ± 5mmHg in falls group vs 13 ± 4mmHg in without falls group; *p *< 0.0001). In a follow-up study [39], maximum decrease in postprandial SBP was an independent risk factor for falls in nursing home residents (risk ratio 1.19, 95% CI 1.15–1.23). In another study [41], a postprandial SBP drop of ≥20mmHg was not associated with increased fall risk (or recurrent falls) in the previous year. A postprandial SBP of 115mmHg or lower was associated with increased risk of falling (OR 3.7, 95% CI 1.3–11.1). These findings suggest that intermittent blood pressure measurements are sensitive enough to diagnose PPH in older adults.

In two (2/16) studies [40, 46], 24-h ambulatory blood pressure monitoring (24-h ABPM) was performed*.* In the first study [40], 24-h ABPM was performed in older admitted adults with syncope/falls and appeared useful to detect PPH. Change in postprandial SBP was greater among patients with syncope/falls compared with patients without history of falls/syncope (4.5 ± 5.7, and 3.8 ± −7.5 vs 1.8 ± 6.6; *p *= 0.015). The number of patients experiencing PPH was significantly higher (23% vs 9%; *p *= 0.03) for those who experienced syncope/falls. In the other study [46], 24-hABPM patterns were evaluated in older hypertensive patients with and without a history of injurious falls. Patients with fall injury compared to patients without fall injury had significantly lower DBP (67.3 ± 7.6 vs 70.7 ± 8.8; *p *> 0.005) and higher pulse pressure (74 ± 14.3 vs 68 ± 13.7; *p *< 0.005), suggesting that performing 24-h ABPM may be of value in fall risk assessment in older hypertensive patients.

### Tests for determining arterial stiffness

We included two studies [54, 55] studying the relationship between arterial stiffness measurements and falls using different techniques. In one study [54], arterial stiffness was determined by measuring supine carotid-femoral pulse wave velocity (PWV) using a semi-automated pulse wave analysis system. Participants with high PWV had an increased fall risk (relative risk: 1.37, 95% 1.06–1.78). In the other study [55], arterial stiffness was measured by determining cardio-ankle vascular index (CAVI). A possible association was found between high arterial stiffness and fall-related injuries, where CAVI above the predicted value was associated with fall-related injuries (OR 3.52, 95% CI 1.03–12.04).

### Tests for heart rhythm assessment

We included nine studies [56–64] on rhythm monitoring in patients with (unexplained) falls. Ambulatory Holter monitoring (AHM) to detect cardiac arrhythmias in fallers was used in three (3/9) studies [57–59]. In one of these [59], AHM detected in 32% of fallers cardiac arrhythmias playing a contributing role. In 18% of non-fallers, abnormal AHM records were found. Suggesting that AHM is useful to detect cardiac arrhythmias. However, in the two other studies [57, 58], AHM failed to discriminate between fallers and non-fallers. An event recorder (reveal) to detect cardiac arrhythmias in fallers was used in one (1/9) study [60]. The authors concluded that the use of an event recorder for 5–7 days may be of value in fallers, as in 34% adjustment of anti-arrhythmic medications or initiation of anti-arrhythmic therapy was required, and in 16.4% consultation by a cardiologist took place. An implantable loop recorder (ILR) to detect cardiac arrhythmias in fallers was used in four (4/9) studies [61–64]. All four studies investigated ILR in patients presenting with unexplained and/or recurrent falls and detected arrhythmias as (attributable) cause of the fall, demonstrating diagnostic value in this population. In one (1/9) study [56], autonomic cardiovascular markers in older fallers were evaluated by using 24h-Holter ECG data (to analyze heart rate variability (HRV), QT dynamicity, and heart rate turbulence (HRT)). There were no differences in HRV and QT dynamicity parameters between the groups, but there was a significant worsening in HRT parameters in fallers compared to non-fallers. The authors concluded that HRT parameters can be used to estimate falls in older adults.

### Carotid sinus massage and tilt table testing

We included one systematic review and meta-analysis [9] and four original studies [65–68] on carotid sinus massage (CSM) and tilt table testing in older fallers. The systematic review by Jansen et al. [9] reports on the association between CSH, VVS, and falls. They found a higher prevalence of CSH in fallers compared to non-fallers, with a wide prevalence range (between 8 and 73%). Most studies performed CSM both in supine and upright (tilted 70°) position. In two studies, CSM was performed in supine position only. In addition, VVS was more common in fallers compared to non-fallers (prevalence range between 3 and 46%). All studies used head-up tilt (HUT) testing.

We included four additional studies on this topic. One (1/4) study [66] reported on the sensitivity and specificity of CSM related to falls history. For participants with any history of falls and a diagnosis of CSH, the sensitivity of CSM was 41%, specificity 64%, positive predictive value 73%, and a negative predictive value of 32%. Another (1/4) study [65] performed CSM (supine and tilted) in hospitalized hip fracture patients and compared these with matched control patients. Patients were categorized to accidental falls (64.7%) or unexplained falls (35.3%). The authors found a significant difference of CSM positivity rate between these groups; 67% of the unexplained fallers had a positive response to CSM versus 18% of accidental fallers (*p *< 0.001). In another (1/4) study [67], reproducibility of repeated (four occasions) CSM was studied in patients with cardioinhibitory (CI)CSH and unexplained/recurrent falls. In most patients, the finding of CICSH was reproducible and a positive finding was more likely shortly after the initial positive response. Half of the participants had CICSH on all four occasions and 17% had a consistent response on the same side in the same position. In the last (1/4) study [68], the effect of head turning in older adults with CSH was studied by continuously measuring BP and heart rate before, during, and after head turning. Head turning induced hypotension (HTIH) (a SBP drop of at least 20mmHg) was observed in 39% of patients and 44% of healthy older adults, irrespective of direction of the head movement. Also, CSH was associated with HTIH (OR 3.5; 95% CI 1.48–8.35).

#### Combined diagnostic approaches

We included seven original studies [69–75] on combined diagnostic approaches. Two (2/7) studies [71, 73] evaluated a 2-day structured multidisciplinary care pathway to evaluate unexplained non-accidental falls and/or syncope in older patients. In the first study [71], this care pathway was developed, and the diagnostic yield was evaluated. This care pathway included among others a 12-lead ECG, orthostatic BP measurements, PPH investigation, 24h-Holter, echocardiogram, cardiologist consultation, and tilt table testing with CSM. Approximately half of the participants diagnosed with syncope also presented with a fall. Of the patients with syncope (*n *= 117), OH was found in 53% of the patients, half had PPH and 24% had a cardiac cause for the syncope. Overall, a final diagnosis could be made in 89% with a large overlap between falls and syncope. The second study of this care pathway [73] showed that for the patients with a final diagnosis of syncope 45% had symptomatic OH/PPH, 44% had a cardiac cause, 21% had reflex syncope and 6% remained unexplained. Overall, the care pathway found in 94% one or more possible explanations for the syncope, where 44% of these patients presented with a fall.

We included four (4/7) studies [69, 70, 72, 75] focusing on diagnostic testing for cardiovascular abnormalities in patients with unexplained falls. One study [75], retrospectively evaluated patients hospitalized with fall-related trauma and/or fracture. The evaluation included a 12-lead ECG, (orthostatic) BP measurements and on indication echocardiography, CSM and prolonged ECG monitoring. Depending on the suspected cause of the fall, patients were categorized into transient loss of consciousness (T-LOC), unexplained fall, or accidental fall. In patients with T-LOC (36%), a probable origin was found in 91% of cases (53% OH, 25% cardiac cause, 6% VVS). In patients with unexplained falls (37%), the prevalence of abnormal ECGs and OH was higher compared to the patients with accidental falls. Another study [72] evaluated a simplified syncope diagnostic protocol, based on the 2009 ESC guideline, in patients with dementia and episodes of T-LOC suspected of syncope or unexplained falls. The protocol included (orthostatic) BP measurements, ECG and CSM in supine position. When no diagnosis was found, additional testing was performed, consisting of 24-h ECG, echocardiography, BP monitoring, ILR implantation (if indicated), and CSM upright and head-up tilt testing. 47% of the participants were evaluated for unexplained falls, of which in 45% syncope was identified as cause of the event with underlying OH in 48%.

In another study [69], patients underwent a cardiovascular investigation with (orthostatic) BP measurements, 12-lead ECG, CSM, and head-up tilt testing. In patients with unexplained and recurrent falls, they found a high probability (in 20 of 26 patients) for finding cardiovascular conditions: in 19% OH, in 73% CSH, in 15% vasovagal hypersensitivity and in 8% arrhythmias. Another study [70] investigated the causes of recurrent drop attacks in older adults with a comprehensive diagnostic evaluation, including a 12-lead ECG, supine and standing CSM, orthostatic BP measurements, 40-min passive head-up tilt, 24-h electrocardiogram, and BP monitoring. Attributable diagnoses were made in 90% of the subjects, of which 53% of the participants a cardiovascular disorder was diagnosed. In the last (1/7) study [74], the presence of cardiovascular autonomic neuropathy (cAN) and its association with falls was by performing a standardized set of autonomic function tests (heart rate variability (HRV), BP assessment during deep breathing, Valsalva maneuver and supine to standing position). The risk of falling was 15 times larger in patients with cAN (OR 15.2, 95% CI 2.28–34.2). The risk of falls in patients with OH was ten times larger (OR 10.7, 95% CI 1.45–29.27). Authors conclude that the sensitivity of autonomic testing was greater than only testing for OH with AS and needs special clinical attention.

#### Single treatment approaches

##### Orthostatic hypotension related treatments

We included one study [76] on midodrine for treating OH in patients with among others unexplained falls and a diagnosis of neurocardiogenic syncope (OH, VVS, or CSH). A positive response (significant reduction or abolition of symptoms; falls not specified) was recorded prospectively. Seventy-one percent reported an abolition or significant reduction across all diagnosis, whereas 29% reported no benefit.

## Discussion

The EM and scoping review show that literature reporting on the fall preventive potential of various cardiovascular diagnostics and treatments is relatively scarce. In this study we updated and extended our previous work [16] by summarizing and visualizing the evidence on the fall risk reducing effect of different diagnostics and treatments in an EM. Majority of the studies included in the EM showed a reduction in fall risk after the intervention. There is evidence for a positive effect of a combined cardiovascular assessment and treatment, drug therapy for OH and for an exercise intervention. There are mixed results for pacemaker implantation. Additionally, in accordance with the WGF recommendations, we found that measuring orthostatic blood pressure (preferably using continuous blood pressure method), 24-h ABPM, Holter monitoring, and ILR, CSM and tilt table testing are effective components [2].

Our review confirmed that a combined and structured assessment is able to identify cardiovascular risk factors in fallers [33, 69–75, 77]. However, studies that report directly on fall risk reduction are scarce. In addition to our earlier scoping review [16], the network meta-analysis by Dautzenberg et al. [15] was included, of which the results suggest that cardiovascular assessment/treatment has fall risk reducing potential. However, in the analyzed studies, the cardiovascular assessments were part of broader (multifactorial) fall preventive interventions. Consequently, it is challenging to determine fall reducing potential of the cardiovascular components separately.

The most common and established cardiovascular risk factor for falls is OH [9, 37]. The WGF [2] recommends including OH management as a component of a multidomain intervention. Whereas many multidomain fall prevention programs have incorporated OH treatment strategies [5, 78, 79], there is a lack of single OH intervention studies in fall prevention. The network meta-analysis by Dautzenberg et al. [15] demonstrated that managing OH was one of the effective components of a multifactorial fall risk assessment in reducing fall rates. For OH treatment as a single intervention, we identified only one study [35], that studied the use of drug treatment (droxidopa) in Parkinson’s patients with neurogenic OH. The post hoc analysis of falls data revealed a statistically significant beneficial effect of droxidopa. However, it is important to recognize that in most older adults, OH is not solely neurogenic, but determined by multiple factors [80, 81]. Other interventions, such as elastic stockings and pharmacological agents as midodrine and fludrocortisone, have demonstrated effectiveness in reducing OH [76, 82, 83]. Although it is likely that reducing OH will contribute to a decreased risk of falls, this has not been studied directly. Furthermore, our findings about the measuring method of OH, are in line with the WGF recommendations. BP measurements should be taken supine to standing [2, 52], intermittent blood pressure measurements with 1-min intervals preferably up to 5 min, and referring for beat-to-beat measurements when OH is suspected but not detected [2]. In addition to existing guidelines and reviews, our review adds that using the 5-s average in continuous blood pressure measurements (Finometer device) needs to be considered [42]. Also, recognizing BP patterns and BP drop rate during the continuously measured orthostatic AS test could assist clinicians in guiding clinical fall risk assessment [51, 53]. However, the exact role of these characteristics needs to be further established in future studies.

CSM and tilt table testing are able to attribute CSH/CSS and VVS as the cause of unexplained and recurrent falls [9]. However, distinguishing between CSH and CSS in older adults can be challenging in clinical practice. Although terminology and definitions differ between studies, there is consensus that the diagnosis of CSS requires both the reproduction of spontaneous symptoms during CSM and the presence of clinical features of syncope compatible with a reflex mechanism (method of symptoms) [2, 14]. CSH refers to an abnormal response to CSM without definite symptom reproduction. A significant number of older adults with unexplained falls exhibit CSH during CSM, suggesting that this may be the underlying mechanism [84]. However, CSH is also observed in older individuals without a history of syncope or falls. Complicating is that older adults may be affected by CSS without prodromal symptoms or have retrograde amnesia for the event [66, 84–86]. The study by Rafanelli et al. [87] found high and similar positivity rates in both tilt testing and CSM among patients with unexplained falls and unexplained syncope, underlying that many unexplained falls are probably cases of VVS or CSS without prodromal symptoms/with retrograde amnesia. Also, interindividual variability in response to CSM has been noted, indicating that there should be a low threshold for repeating CSM in patients suspected of having CSH (as with unexplained falls) [67]. Additionally, our review highlights that head turning appears to be an important common trigger for hypotension in patients with CSH [68]. Although these results need to be replicated, incorporating head turning into the CSM protocol and counseling patients on the effect of head turning could be easily implemented.

In line with the WGF recommendation [2], the evidence concerning pacemaker implantation in patients with recurrent falls and CICSH remains inconclusive. The included meta-analysis on this topic showed a significant reduction in rate of falls, but not in risk of falls after pacemaker implantation [36]. The three RCTs [88–90] included in this meta-analysis present contradictory results. The trial [90] that reported a positive effect of pacemaker implantation on falls was a single-center study, in a long-established clinical facility with clear pathways for referral, evaluation, and management of older people with syncope and falls. The trial that followed this study [88], which was not able to show a positive effect, was a multicenter study and also included centers without systems/pathways for managing older people with syncope and falls and patient characteristics differed between the study populations (e.g., were older, more comorbid cardiovascular disease, cognitive frailer, and more moderate response to carotid sinus pressure). Expanding the intervention to a more diffuse population could make the intervention less successful. Surprisingly, in this study, there was a significant reduction in falls in both groups after device (pacemaker or ILR) implantation, suggesting a placebo effect. The other (non-randomized) studies [28–32] we included on this topic all show a reduction in falls after pacemaker implantation but have high risk of bias by lacking control groups, having an observational study design and collecting data on falls retrospectively.

A potentially valuable feature of continuously blood pressure measurements, as derived from our review that is not yet standard practice, is BPV (fluctuations in BP). Where individuals have apparently normal/comparable BP levels, there could be abnormal BP fluctuations [91]. Literature published so far mainly describes variability in intermittent BP measurements rather than in continuous BP time series [92] and literature on BPV and falls is scarce. We included only two studies that focused on BPV and falls [43, 45]. The findings indicated that reduced standing beat-to-beat BPV during AS was associated with increased risk of falls. Reduction in BPV in fallers suggests a potential impairment in BP control reactivity in the upright posture, which could contribute to susceptibility to falling [45]. However, these results are exploratory in nature and further research is necessary to establish the relevance of BPV in fall prevention.

Another potential diagnostic measure for cardiovascular fall risk, not standardly addressed in clinical practice, is arterial stiffness [93]. Arterial stiffness is associated with greater risk of cardiovascular events (e.g., stroke), cognitive decline, and OH [93–95]. There are several non-invasive methods to assess arterial stiffness, as PWV and CAVI [54, 55]. Based on our review, these diagnostics might be of interest for fall risk assessment. However, both studies were cross-sectional in nature and the exact value of these diagnostics within fall prevention remains unclear.

### Strengths and limitations

Our EM and scoping review have several strengths. First, we conducted a comprehensive literature search in collaboration with experienced medical librarian to capture all relevant published literature on the impact of cardiovascular diagnostics and treatments for the prevention of falls in older adults. We summarized and visualized the available evidence in an EM and the scoping review methodology allowed us for a broad examination of the literature. As such, our summary of the literature may be of value for developing structured, standardized care pathway. There are also several limitations. Firstly, despite this large number of references, the number of studies reporting on effectiveness of a diagnostic/intervention on falls risk is still scarce. We performed a single screening approach for the largest part of the dataset. Although there was near-complete concordance between the two reviewers, this may have introduced a risk of excluding relevant publications. Secondly, the differences in the study populations, study designs, data gathering, definitions, and used methods (e.g., AS for 3 min in most studies, but also 1 min AS was used) make it difficult to compare studies and draw firm conclusions. Thirdly, we did not include multifactorial intervention studies since they do not report on the fall risk reducing effect of the individual cardiovascular interventions.

### Evidence gaps and future research

We identified several research gaps for the diagnostic value of the individual components of the cardiovascular fall risk assessment. For example, the diagnostic yield of examinations like 24h-ECG monitoring, echocardiography and treatment like anti-arrhythmics, valve replacement and single OH treatments are currently unknown. For future review projects, it is also interesting to include cerebrovascular diagnostics, e.g., the role of brain imaging (e.g., CT or MRI) in fall prevention. Also, diagnostics in which cerebral perfusion is displayed could be interesting for fall prevention. For example, near-infrared spectroscopy (NIRS), which uses near-infrared light measuring through the skull to detect changes in oxygenated and deoxygenated hemoglobin as a measure for cerebral perfusion [96]. NIRS might be a better reflection of orthostatic symptoms than BP measurements, due to the direct measurement of cerebral oxygenation [96].

Also, for syncope units it has been demonstrated that they reduce hospitalization and reduces cost of syncope [97]. Considering the large overlap between syncope and (unexplained) falls, this could also apply for fall clinics that perform a structured cardiovascular assessment/care pathway. However, the actual reduction of falls of these structured approaches first needs to be further studied in randomized controlled trials.

## Conclusion

In this study, we summarized available evidence on the impact of cardiovascular diagnostics and treatments in the prevention of falls among older adults, visualized by EM methodology together with a detailed scoping review. Cardiovascular disorders are established fall risk factors and fall prevention guidelines recommend incorporating cardiovascular assessments as part of a multifactorial approach [2]. However, the number of studies investigating effectiveness of individual cardiovascular diagnostics and treatments in reducing falls is limited, and there is still uncertainty regarding the optimal diagnostic approach. As recommended by the WGF prevention, the cornerstone should always initially be cardiac history taking, auscultation, lying and standing orthostatic blood pressure measurements and a surface 12-lead electrocardiogram [2]. Additionally, further assessments similar to those recommended for syncope should be considered, following the ESC syncope guideline [14]. We identified several diagnostics that require further investigation regarding their role in fall prevention, as blood pressure variability, arterial stiffness, and head turning. Our results can be used to optimize (or develop) an evidence-based care pathway for fall prevention, but there is a need for a well-designed trial to evaluate the efficacy and optimal content of a cardiovascular evaluation and intervention for reducing falls among older adults.

### Supplementary information

ESM 1 (DOCX 230 kb)
