# Supplementary material for: The impact of cardiovascular diagnostics and treatments on fall risk in older adults: a scoping review and evidence map
Source: GeroScience. 2023 Oct 21;46(1):153–69. doi: 10.1007/s11357-023-00974-4 (PMC10828261; doi:10.1007/s11357-023-00974-4)
Supplement: Supplementary file 1 — (DOCX 230 kb) [file 11357_2023_974_MOESM1_ESM.docx]

**Appendices**

[Appendix A. Table 1 PRISMA-ScR Checklist 2](#_Toc103433093)

[Appendix B. Table 1 Search strategy for Medline 4](#_Toc103433094)

[Appendix B. Table 2 Search strategy for Embase 5](#_Toc103433094)

[Appendix C. Critical appraisal of studies included in the evidence map 7](#_Toc103433095)

- [Table 1 Critical appraisal for RCTs (RoB 2 checklist) 7](#_Toc103433095)
- [Table 2 Critical appraisal for non-RCTs of interventions (ROBINS-I checklist) 7](#_Toc103433095)
- [Table 3 Critical appraisal for observational studies (JBI critical appraisal tool) 7](#_Toc103433095)
- [Table 4 Critical appraisal for systematic reviews (AMSTAR 2 checklist) 8](#_Toc103433095)

[Appendix D. Table 1 Overview of studies on combined cardiovascular diagnostics and treatments 9](#_Toc103433096)

[Appendix D. Table 2 Overview of studies on pacemaker intervention 11](#_Toc103433112)

[Appendix D. Table 3 Overview of studies on orthostatic hypotension-related treatments 12](#_Toc103433112)

[Appendix D. Table 4 Overview of studies on cardiovascular exercise intervention 13](#_Toc103433112)

[Appendix D. Table 5 Overview of studies on (orthostatic) blood pressure measurements 14](#_Toc103433112)

[Appendix D. Table 6 Overview of studies on arterial stiffness measurements 19](#_Toc103433112)

[Appendix D. Table 7 Overview of studies focusing on heart rhythm assessments 20](#_Toc103433112)

[Appendix D. Table 8 Overview of studies focusing on carotid sinus massage and tilt table testing 22](#_Toc103433112)

[References 24](#_Toc103433133)

**Appendix A**

**Table 1.** **Preferred Reporting Items for Systematic reviews and Meta-Analyses extension for Scoping Reviews (PRISMA-ScR) Checklist**

| **SECTION** | **ITEM** | **PRISMA-ScR CHECKLIST ITEM** | **REPORTED ON PAGE #** |
| --- | --- | --- | --- |
| **TITLE** | | | |
| Title | 1 | Identify the report as a scoping review. | 1 |
| **ABSTRACT** |  |  |  |
| Structured summary | 2 | Provide a structured summary that includes (as applicable): background, objectives, eligibility criteria, sources of evidence, charting methods, results, and conclusions that relate to the review questions and objectives. | 3 |
| **INTRODUCTION** | | | |
| Rationale | 3 | Describe the rationale for the review in the context of what is already known. Explain why the review questions/objectives lend themselves to a scoping review approach. | 4,5 |
| Objectives | 4 | Provide an explicit statement of the questions and objectives being addressed with reference to their key elements (e.g., population or participants, concepts, and context) or other relevant key elements used to conceptualize the review questions and/or objectives. | 4,5 |
| **METHODS** | | | |
| Protocol and registration | 5 | Indicate whether a review protocol exists; state if and where it can be accessed (e.g., a Web address); and if available, provide registration information, including the registration number. | 5 |
| Eligibility criteria | 6 | Specify characteristics of the sources of evidence used as eligibility criteria (e.g., years considered, language, and publication status), and provide a rationale. | 5, 6 |
| Information sources* | 7 | Describe all information sources in the search (e.g., databases with dates of coverage and contact with authors to identify additional sources), as well as the date the most | 6 |
| Search | 8 | Present the full electronic search strategy for at least 1 database, including any limits used, such that it could be repeated. | 6 |
| Selection of sources of evidence† | 9 | State the process for selecting sources of evidence (i.e., screening and eligibility) included in the scoping review. | 6 |
| Data charting process‡ | 10 | Describe the methods of charting data from the included sources of evidence (e.g., calibrated forms or forms that have been tested by the team before their use, and whether data charting was done independently or in duplicate) and any processes for obtaining and confirming data from investigators. | 6 |
| Data items | 11 | List and define all variables for which data were sought and any assumptions and simplifications made. | 6 & Appendix D |
| Critical appraisal of individual sources of evidence§ | 12 | If done, provide a rationale for conducting a critical appraisal of included sources of evidence; describe the methods used and how this information was used in any data synthesis (if appropriate). | 7 |
| Synthesis of results | 13 | Describe the methods of handling and summarizing the data that were charted. | 7 |
| **RESULTS** | | | |
| Selection of sources of evidence | 14 | Give numbers of sources of evidence screened, assessed for eligibility, and included in the review, with reasons for exclusions at each stage, ideally using a flow diagram. | 7-10 |
| Characteristics of sources of evidence | 15 | For each source of evidence, present characteristics for which data were charted and provide the citations. | 10-11, 13 |
| Critical appraisal within sources of evidence | 16 | If done, present data on critical appraisal of included sources of evidence (see item 12). | 11 |
| Results of individual sources of evidence | 17 | For each included source of evidence, present the relevant data that were charted that relate to the review questions and objectives. | 11-19 |
| Synthesis of results | 18 | Summarize and/or present the charting results as they relate to the review questions and objectives. | 9-19 |
| **DISCUSSION** | | | |
| Summary of evidence | 19 | Summarize the main results (including an overview of concepts, themes, and types of evidence available), link to the review questions and objectives, and consider the relevance to key groups. | 20-23 |
| Limitations | 20 | Discuss the limitations of the scoping review process. | 23 |
| Conclusions | 21 | Provide a general interpretation of the results with respect to the review questions and objectives, as well as potential implications and/or next steps. | 24 |
| **FUNDING** | | | |
| Funding | 22 | Describe sources of funding for the included sources of evidence, as well as sources of funding for the scoping review. Describe the role of the funders of the scoping review. | 25 |

From Tricco, AC, Lillie, E, Zarin, W, O'Brien, KK, Colquhoun, H, Levac, D, Moher, D, Peters, MD, Horsley, T, Weeks, L, Hempel, S et al. PRISMA extension for scoping reviews (PRISMA-ScR): checklist and explanation. Ann Intern Med. 2018,169(7):467-473. doi:10.7326/M18-0850

**Appendix B**

**Table 1. Search strategy for Medline**

**Ovid MEDLINE(R) ALL <1946 to June 29, 2022> Search date: 30 June 2022**

| **Search** | **Query** | **Items found** |
| --- | --- | --- |
| 1 | Geriatric assessment/ or frail elderly/ or exp aged/ or middle aged/ or exp nursing homes/ or "homes for the aged"/ | 5505589 |
| 2 | (older person? or older patient? or middle aged or seniors or senior citiz* or elder or elders or elderly or geriatric* or frailty or postmenopausal women or community-dwelling or nursing home? or resident* or old* people or old* person? or old* patient? or old* client? or old* adult?).ab,kf,ti. | 828936 |
| 3 | (geriatr* or age or aging or elderl*).in,jw. | 299530 |
| 4 | or/1-3 [persons 50 yrs or older] | 5955255 |
| 5 | (cardiovascular adj3 (assessment or evaluation? or intervention?)) or (Blood Pressure adj3 (monitoring or self or ambulatory or home))).ab,kf,ti. | 27047 |
| 6 | (Orthostatic hypotension or postural hypotension or Tilt-table test or head-down tilt or Carotid sinus massage or Electrocardiogram or ECG or Electrocardiograph or Transthoracic Echocardiography or Cross-Sectional Echocardiography or Cross Sectional Echocardiography or "m-Mode" or Contrast Echocardiography or 2D Echocardiography or Two Dimensional Echocardiography or "2-D Echocardiography" or Dynamic Electrocardiography or Ambulatory Electrocardiographic Monitoring or Ambulatory Electrocardiography or Holter Electrocardiography or Holter Monitoring or Loop recorder).ab,kf,ti. | 141162 |
| 7 | (pacemaker? or Cardiac Valve Annuloplast* or cardiac pacing or Valvular Annuloplasty or Valvular Annuloplast* or Heart Valve Annuloplasty or Cardiac Valve Annulus Repair or heart Valve Annulus Repair or Cardiac Valve Annular Repair or Heart Valve Annular Repair or Cardiac Valve Annular Reduction or Cardiac Valve Annulus Shortening or Cardiac Valve Annulus Reduction or Mitral Valve Annuloplast* or Mitral Valve Annulus Repair or (Coronary adj3 Balloon) or Coronary Angioplasty).ab,kf,ti. | 58227 |
| 8 | ((exercise adj3 intervention*) or (fall adj3 program*)).mp. | 13279 |
| 9 | review.pt. | 3007566 |
| 10 | 8 and 9 | 2854 |
| 11 | (sinus carotid hypersensitivity or vasovagal collapse or postprandial hypotension or heart failure or cardiac rythm disorder or structural cardiac disorder?).ab,kf,ti. | 202655 |
| 12 | ((evaluat* or scor* or assess*) and (TAVI or Transcatheter Aortic Valve Implant* or CABG or Coronary artery bypass graft* or midodrine or cardiovascular drug? or antiarrhythmic drug? or anti arrhytmic drug? or diuretic? or beta blocker? or calcium channel blocker? or ACE inhibitor? or angiotensin receptor blocker? or vasodilator? or vasoactive drug? or Cardio fitness)).mp. | 86163 |
| 13 | ((heart or cardi*) and (physical examination or (tak* adj3 history) or ablation or defribillator or fludrocortisone or tca? or ephedrine or desmopressin or DDAVP or octreotide or erythropoietin or nonsteroidal anti-inflammatory agent? or salt intake? or Abdominal binder? or support stocking? or Elastic compression therapy or tai chi or Education or lifestyle or prodromal symptoms? or (head adj3 tilt*) or (head adj3 elevat*) or alcohol intake or fluid intake or lower body muscle)).ab,kf,ti. | 92578 |
| 14 | or/5-7,10-13 [cardiovascular evaluation \| -interventions] | 554028 |
| 15 | accidental falls/ or exp syncope/ | 40906 |
| 16 | (fall? or fell or falling or fallen or faller or stumble? or stumbling or stumbles or slip or slips or slipping or slipped or trip or tripped or syncope or TLOC or "Transient loss of consciousness").ab,kf,ti. | 302145 |
| 17 | 15 or 16 | 313003 |
| 18 | and/4,14,17 | 9711 |
| 19 | (ISRCTN11674947 or NCT01037426 or myfait or (safe pace and (study or trial))).ab,kf,ti. | 10 |
| 20 | 18 or 19 | 9715 |

**Table 2.** **Search strategy for Embase**

**Ovid Embase Classic+Embase <1947 to 2022 June 29> Search date: 30 June 2022**

| **Search** | **Query** | **Items found** |
| --- | --- | --- |
| 1 | *Geriatric assessment/ or exp *aged/ or *middle aged/ or exp *nursing homes/ or *"home for the aged"/ | 98853 |
| 2 | (older person? or older patient? or middle aged or seniors or senior citiz* or elder or elders or elderly or geriatric* or frailty or postmenopausal women or community-dwelling or nursing home? or resident* or old* people or old* person? or old* patient? or old* client? or old* adult?).ab,kw,ti. | 1139363 |
| 3 | (geriatr* or age or aging or elderl*).in,jx. | 427334 |
| 4 | or/1-3 [persons 50 yrs or older] | 1421301 |
| 5 | ((cardiovascular adj3 (assessment or evaluation? or intervention?)) or (Blood Pressure adj3 (monitoring or self or ambulatory or home))).ab,kw,ti. | 41698 |
| 6 | (Orthostatic hypotension or postural hypotension or Tilt-table test or head-down tilt or Carotid sinus massage or Electrocardiogram or ECG or Electrocardiograph or Transthoracic Echocardiography or Cross-Sectional Echocardiography or Cross Sectional Echocardiography or "m-Mode" or Contrast Echocardiography or 2D Echocardiography or Two Dimensional Echocardiography or "2-D Echocardiography" or Dynamic Electrocardiography or Ambulatory Electrocardiographic Monitoring or Ambulatory Electrocardiography or Holter Electrocardiography or Holter Monitoring or Loop recorder).ab,kw,ti. | 242211 |
| 7 | (pacemaker? or Cardiac Valve Annuloplast* or Valvular Annuloplasty or Valvular Annuloplast* or Heart Valve Annuloplasty or Cardiac Valve Annulus Repair or heart Valve Annulus Repair or Cardiac Valve Annular Repair or Heart Valve Annular Repair or Cardiac Valve Annular Reduction or Cardiac Valve Annulus Shortening or Cardiac Valve Annulus Reduction or Mitral Valve Annuloplast* or Mitral Valve Annulus Repair or (Coronary adj3 Balloon) or Coronary Angioplasty).ab,kw,ti. | 83236 |
| 8 | ((exercise adj3 intervention*) or (fall adj3 program*)).mp. | 17953 |
| 9 | review.pt. | 2967362 |
| 10 | 8 and 9 | 2868 |
| 11 | (sinus carotid hypersensitivity or vasovagal collapse or postprandial hypotension or heart failure or cardiac rythm disorder or structural cardiac disorder?).ab,kw,ti. | 340541 |
| 12 | ((evaluat* or scor* or assess*) and (TAVI or Transcatheter Aortic Valve Implant* or CABG or Coronary artery bypass graft* or midodrine or cardiovascular drug? or antiarrhythmic drug? or anti arrhytmic drug? or diuretic? or beta blocker? or calcium channel blocker? or ACE inhibitor? or angiotensin receptor blocker? or vasodilator? or vasoactive drug? or Cardio fitness)).mp. | 172139 |
| 13 | ((heart or cardi*) and (physical examination or (tak* adj3 history) or ablation or defribillator or fludrocortisone or tca? or ephedrine or desmopressin or DDAVP or octreotide or erythropoietin or nonsteroidal anti-inflammatory agent? or salt intake? or Abdominal binder? or support stocking? or Elastic compression therapy or tai chi or Education or lifestyle or prodromal symptoms? or (head adj3 tilt*) or (head adj3 elevat*) or alcohol intake or fluid intake or lower body muscle)).ab,kw,ti. | 157179 |
| 14 | or/5-7,10-13 [cardiovascular evaluation \| -interventions] | 917578 |
| 15 | *falling/ or *faintness/ | 17681 |
| 16 | (fall? or fell or falling or fallen or faller or stumble? or stumbling or stumbles or slip or slips or slipping or slipped or trip or tripped or syncope or TLOC or "Transient loss of consciousness").ab,kw,ti. | 436076 |
| 17 | 15 or 16 | 437438 |
| 18 | and/4,14,17 | 4893 |
| 19 | (ISRCTN11674947 or NCT01037426 or myfait or (safe pace and (study or trial))).ab,cn,kw,ti. | 21 |
| 20 | 18 or 19 | 4905 |

**Appendix C. Critical appraisal of studies included in evidence map**

**Table 1.** **Critical appraisal for RCTs (RoB 2 checklist** [1]**)**

| Studies | 1.Random sequence intervention | 2.Random sequence patients | 3.Blinding patients and practitioners | 4.Blinding effect assessors | 5.Groups comparable | 6.Complete follow-up | 7.Analyzed included patients | 8.Treated equally | 9.Selective reporting | 10.Sponsors influence |
| --- | --- | --- | --- | --- | --- | --- | --- | --- | --- | --- |
| Hauser 2016 [2] | + | + | + | ? | + | + | + | + | - | + |

**Checking items**: Item 1: Was the allocation of the intervention to the patients randomized? Item 2: The person who includes patients should not be aware of the randomization sequence. Was that the case here? Item 3: Were the patients and the practitioners blinded for the treatment? Item 4: Were the effect assessors blinded for treatment? Item 5: Were the groups comparable at the beginning of the trial? If not: has this been corrected in the analyzes? Item 6: Is a complete follow-up available from a sufficient proportion of all participants? If not: is selective loss-to- follow-up sufficiently excluded? Item7: Have all the included patients been analyzed in the group in which they were randomized? Item 8: Have the groups been treated equally, apart from the intervention? Item 9: Is selective publication of results sufficiently excluded? Item 10: Is unwanted influence of sponsors sufficiently excluded? **Judgment:** ‘+’ sign with green color, indicates low risk of bias; ‘- ‘sign with red color, indicates high risk of bias; ‘?’ sign with orange color, indicates unclear risk of bias. RoB: risk of bias

**Table 2.** **Critical appraisal for non-RCTs of interventions (ROBINS-I checklist** [3]**)**

| Studies | 1.Confounding bias | 2. Participants selection bias | 3.Interventions classification bias | 4.Intended interventions deviations bias | 5. Missing data bias | 6.Outcomes measurement bias | 7. Reported result selection bias |
| --- | --- | --- | --- | --- | --- | --- | --- |
| Crilley  1997 [4] | *x* | + | + | + | *x* | - | + |
| Krasniqi 2012 [5] | *x* | *x* | - | + | - | - | + |
| Jansen  2015 [6] | - | - | + | + | + | - | + |
| Brenner 2017 [7] | + | + | + | + | - | - | + |

**Checking items:** Item 1: bias due to confounding; Item 2: bias due to selection of participants; Item 3: bias in classification of interventions; Item 4: bias due to deviations from intended interventions; Item 5: bias due to missing data; Item 6: bias in measurement of outcomes; and Item 7: bias in selection of the reported result. **Judgement:** ‘+’ sign with green color, indicates low risk of bias; ‘- ‘sign with orange color, indicates moderate risk of bias; ‘*×*’ sign with red color, indicates high risk of bias. ROBINS-I: risk of bias in non-randomized studies of interventions

**Table 3.** **Critical appraisal for observational studies (JBI critical appraisal tool** [8]**)**

| Studies | 1.Similar groups | 2.Similar exposure | 3.Valid exposure | 4.Confounding | 5.Confounding strategies | 6.Outcome | 7.Measure outcomes | 8.Follow up | 9.Follow up complete | 10.Incomplete strategies | 11.Statistical analysis |
| --- | --- | --- | --- | --- | --- | --- | --- | --- | --- | --- | --- |
| Bexton  1997 [9] | + | N/A | ? | N/A | N/A | + | ? | N/A | ? | ? | ? |
| Karunaratne 2002 [10] | N/A | + | + | - | - | + | - | + | + | - | - |
| Genta  2017 [11] | - | + | + | - | - | ? | + | + | ? | ? | + |

**Checking items**: Item 1: Were the two groups similar and recruited from the same population? Item 2: Were the exposures measured similarly to assign people to both exposed and unexposed groups? Item 3: Was the exposure measured in a valid and reliable way? Item 4: Were confounding factors identified? Item 5: Were strategies to deal with confounding factors stated? Item 6: Were the groups/participants free of the outcome at the start of the study (or at the moment of exposure)? Item 7: Were the outcomes measured in a valid and reliable way? Item 8: Was the follow up time reported and sufficient to be long enough for outcomes to occur? Item 9: Was follow up complete, and if not, were the reasons to loss to follow up described and explored? Item 10: Were strategies to address incomplete follow up utilized? Item 11: Was appropriate statistical analysis used? **Judgment**: ‘+’ sign with green color, indicates low risk of bias; ‘- ‘sign with red color, indicates high risk of bias; ‘?’ sign with orange color, indicates unclear risk of bias; ‘N/A ‘sign with grey color, indicates not applicable. JBI: Joanna briggs institute

**Table 4. Critical appraisal for systematic reviews (AMSTAR 2 checklist** [12]**)**

| Studies | 1.Report protocol | 2.Literature search strategy | 3.Excluded studies list | 4.Assessing risk of bias (RoB) | 5.Statistical methods | 6.Account for RoB in individual studies | 7.Publication bias |
| --- | --- | --- | --- | --- | --- | --- | --- |
| Gillespie 2012 [13] | + | + | + | + | + | + | + |
| Dautzenberg 2021 [14] | + | + | + | + | + | + | + |

**Checking items**: Item 1: Did the report of the review contain an explicit statement that the review methods were established prior to conduct of the review and did the report justify any significant deviations from the protocol? Item 2: Did the review authors use a comprehensive literature search strategy? Item 3: Did the review authors provide a list of excluded studies and justify the exclusions? Item 4: Did the review authors use a satisfactory technique for assessing the risk of bias (RoB) in individual studies that were included in the review? Item 5: If meta-analysis was justified did the review authors use appropriate methods for statistical combination of results? Item 6: Did the review authors account for RoB in individual studies when interpreting/ discussing the results of the review? Item 7: If they performed quantitative synthesis did the review authors carry out an adequate investigation of publication bias (small study bias) and discuss its likely impact on the results of the review? **Judgement**: ‘+’ sign with green color, indicates low risk of bias; ‘- ‘sign with red color, indicates high risk of bias; ‘?’ sign with orange color, indicates unclear risk of bias. AMSTAR: A MeaSurement Tool to Assess systematic Reviews

**Appendix D. Study characteristics of studies included in scoping review and evidence map**

| **Table 1. Overview of studies on combined cardiovascular diagnostics and treatments** | | | | | | | | | | |
| --- | --- | --- | --- | --- | --- | --- | --- | --- | --- | --- |
| **Author, year** | **Design & data gathering** | **N** | **Mean Age (SD) or median (range)** | **Population** | **Setting** | **CV interventions (diagnostics and/or treatments)** | **FU time** | **Outcome (measurement)** | | **Results** |
|  |  |  |  |  |  |  |  | **Fall-related** | **Others** |  |
| Dautzenberg,  2021  [14] | Systematic review and network meta-analysis | 1592 participants (5 studies concerning CV assessment and OH management) | N/A | age ≥65 years | Community dwelling | Category: Basic falls risk assessment containing vital signs, ECG, loop recorder, pacemaker interrogation, medication review  Surgery: e.g. pacemaker implantation.  Management of OH: Wearing elastic stockings, rising slowly, sleeping in a bed with head raised, pharmacological interventions | N/A | Primary outcomes: number of fallers and number of fall-related fractures. Secondary outcomes: number of repeated fallers, number of hip fractures, falls rate, and fracture rate. | N/A | The single intervention basic falls risk assessment is most strongly associated with reduction in number of fall-related fractures (RR 0.60; 95% CI 0.39-0.94).  The single intervention Management of OH was not associated with reducing fall risk, but OH-treatment was one of the effective components (in different combinations) in reducing falls of multidomain interventions. |
| Perego, 2021  [15] | Cohort, retrospective | 100 | 78.4  (4.5) | Patients aged ≥65 years admitted to the SACU with a diagnosis of fall-related trauma and/or fracture | SACU | Evaluation included in-depth medical interview, physical examination, a 12-lead ECG, arterial BP supine and during standing, routine blood test, MMSE, CIRSc. Interview with eyewitness of the fall, and specific cardiological tests, e.g. transthoracic echocardiography; prolonged ECG monitoring; and CSM, based on the clinical judgment. | 1 year | Retrospective: Suspected cause of the fall: T-LOC, unexplained fall (UF), or accidental fall (AF) | Suspected cause of the T-LOC | 36 were categorized as T-LOC, 37 as UF and 27 as AF. Of the 36 patients with T-LOC, a probable origin was identified in most cases (n = 33, 91%). 19 subjects (53%) had OH, 9 (25%) a cardiac relevant disturbance, 2 (6%) a reproduced vasovagal syncope.  In 8 patients (19%) a major cardiac disease was identified (e.g., paroxysmal atrial fibrillation, ventricular tachycardia, severe aortic stenosis). |
| Romagnolo, 2019  [16] | Cohort, prospective | 50 | 65.1  (9.2) | Patients with Parkinson’s disease, age between 30 and 85 years on stable doses of dopaminergic treatment for at least 4 weeks. | Movement Disorder Center | Standardized battery of autonomic tests: HRV and BP assessment during deep breathing, Valsalva maneuver, and laying to standing. | 12 mon. | Prospective:  Number of falls assessed during follow-up visits.  Secondary: history of falls. | Presence of cAN and its association with falls | During follow-up: 56% of patients (28/50) had at least one fall. 42.9% (12/28) reported their first fall during the follow up. The remaining 57.1% (16/28) were already classified as fallers at baseline. Adjusted there was a 15.2 greater ratio of falls in patients with cAN (OR 15.2; 95% CI 2.28–34.20, p = 0.011) and a 10.7 greater ratio of falls in patients with OH (OR 10.70; 95% CI 1.45–29.27; p=0.020). |
| De Ruiter, 2018  [17] | Observational series, retrospective | 117 | 80.0  (6.5) | Patients referred for evaluation of unexplained falls and/or T-LOC with a final conclusion of syncope. | Fall and syncope day clinic | 2 day structured diagnostic program included a comprehensive geriatric assessment, 12-lead ECG, BP measurements for OH and PPH, 24h-Holter, echocardiogram, tilt testing with CSM. |  | Retrospective:  Unexplained falls as indication for evaluation | Category, number, and accuracy of diagnoses resulting in syncope.  Secondary outcomes: reliability of the medical history and the number of ECG abnormalities. | Symptomatic OH/PPH was present in 45%, 44% had cardiac cause, 21% had reflex syncope, 6% remained unexplained.  In 25% a useful eyewitness was available. 64% of ECGs showed relevant abnormalities: 26% suggestive of cardiac syncope, of which 20% showed an indication for device implantation. 50% of syncope patients presented only with falls. |
| Ungar, 2016  [18] | Observational series, prospective | 357 | 83.5  (6.5) | Individuals aged ≥65 years with dementia and one or more episodes of T-LOC of suspected syncopal nature or unexplained falls during the previous 3 months | Multicenter. Geriatric departments. | A simplified syncope diagnostic protocol based on ESC guidelines: detailed history taking, physical examination, BP, ECG. CSM in supine position, orthostatic BP in 1 and 3 min after standing. When reliable diagnosis could not be made after initial evaluation and suspected cardiac, nonreflex or unexplained syncope were referred to syncope unit for 2nd level evaluation: 24h-ECG, echocardiography, BP monitoring, ILR implantation, CSM in upright and head-up tilt testing (using nitroglycerine and continuous BP recording). | N/A | Retrospective: Unexplained falls as indication for evaluation. | Causes of T-LOC suspected to be responsible for syncope and unexplained falls | 181 (50.7%) had been referred for T-LOC suspected to be syncope, 166 (46.5%) for unexplained falls, 10 (2.8%) for both.  Referred for unexplained falls: an initially suspected diagnosis of syncope was confirmed in 158 (87.3%), and syncope was identified as the cause of the event in 75 (45.2%). OH, was the cause of the event in 117 of 242 (48.3%) participants with a final diagnosis of syncope. |
| Jansen, 2015  [6] | Cohort, prospective | 15 | 75.0  (7.0) | Individuals ≥ 65 years with one or more falls in past year referred to a falls clinic. | Falls clinic | A comprehensive CV assessment included structured history taking, ECG, echocardiography, tilt table testing with continuous BP monitoring. Followed by a multidisciplinary evaluation with treatment advice (cardiologist and geriatrician) | 6 mon. | Prospective  Secondary: number of falls | Primary: diagnosis of one or more additional  CV conditions that contributed to a fall. | 47% (7/15) of patients were diagnosed with a CV abnormality contributing to the fall. 33% of patients had a fall incident during follow-up. One injurious fall. CVDs contributing to a fall: initial & delayed OH, drug-induced hypotension, CSS. |
| Wold, 2015  [19] | Cohort, prospective | 262 | 79.6 (6.5) | Patients ≥ 65 years with unexplained fall and/or T-LOC | Fall and syncope day clinic | 2 day structured diagnostic program included a comprehensive geriatric assessment, 12-lead ECG, BP measurements for orthostatic and postprandial hypotension, 24h Holter, echocardiogram, tilt testing with CSM. | N/A | Retrospective:  Unexplained falls as indication for evaluation | Diagnostic yield of the care pathway: cause of the fall/T-LOC or conclusions leading to a treatment. | 44 (18%) were referred for evaluation of syncope while in 117 patients a syncope was diagnosed. Of these 50 presented with a fall. 53% had OH, 50% postprandial hypotension. 24% cardiac cause for syncope. |
| Parry, 2005  [20] | Observational series, cross sectional | 93 | 77.4 (range 55.0-92.0) | Patients ≥ 55 years with three or more unexplained drop attacks (including the index event) in the six months before enrollment. | ED and falls and syncope facility. | Detailed personal and witness history, physical examination included assessment of neurological, locomotor and cardiovascular systems per guidelines of falls and syncope. All subjects underwent 12-lead ECG, bilateral supine and erect CSM and morning orthostatic BP measurements. In case of diagnostic uncertainty, 40-min passive head-up tilt with or without glyceryl trinitrate provocation and 24-hour ambulatory electrocardiogram and BP monitoring. If indicated: echocardiography. | 18 mon. | Retrospective:  Drop attacks (unexplained falls) as indication for further evaluation. | Attributable diagnoses for falls/drop attack, association of symptoms with a positive test: CSH (CI-, VD-, or mixed type), OH, VVS | Attributable diagnoses were made in 90% of subjects. The largest group was cardiovascular disorders, which accounted for attributable diagnosis in 53% of subjects.  37 (40%) CHS (n=35 CI/mixed, n=2 VD). Head-up tilt test (HUTT) in 71% (n=66): VVS was diagnoses in three (3%), with five (5%) being false positive. OH in 5 (5%).  12-lead ECG: 12% having features of ischemic heart disease, 9% sinus bradycardia and 8% first degree atrioventricular block, 2% atrial fibrillation.  24h-ECG in 66% (n=61) of subjects (in all with sinus bradycardia or features of ischemic heart disease): 4 showed any diagnostic feature.  24-h ABPM: in 48% (n=45), 17% showing reversal of normal diurnal variation in BP and 4% having hypertension. |
| Davies, 1996  [21] | Observational series, cross-sectional | 188 | 79.0  (8.0) | Patients ≥ 65 years presenting with falls. Those with unexplained (UF) or recurrent falls (RF) underwent a detailed assessment. | ED | CV investigation: 12-lead ECG, orthostatic BP measurement, CSM (supine and during tilt) and head-up tilt testing (70 degrees for 30min). | N/A | Retrospective: Unexplained/recurrent falls as indication for further evaluation | CV abnormalities: among other diagnose of OH, CSH, VVS, arrhythmia | 26 with UF and RF were fully investigated. CV findings were present in 20/26 patients: OH (19%), CSH (73%), vasovagal hypersensitivity (15%) and arrhythmia (8%). During CSM 7 patients had witnessed LOC, but denied this, demonstrating amnesia for LOC. |

**Abbreviations:** ABPM: ambulatory blood pressure monitoring, AF: accidental fall, BP: blood pressure, cAN : cardiovascular autonomic neuropathy, CI: confidence interval, CICSH: cardioinhibitory subtype of carotid sinus hypersensitivity, CIRS: cumulative illness rated state, CIRS-CI: cumulative illness rated state-comorbidity index, CSM: carotid sinus massage, CSS: carotid sinus syndrome, CSH: carotid sinus hypersensitivity, CV: cardiovascular, CVD: cardiovascular disease/disorder, Dep.: Department, ECG: electrocardiogram, ED: emergency department, FU: follow-up, HR: hazard ratio, HRV: heart rate variability, HUTT: head-up tilt test, LOC: transient loss of consciousness, MMSE: Mini Mental State Examination (range 0–30, where 30 is normal), mon.: months, N/A: not available/applicable, OH: orthostatic hypotension, OR: odds ratio, PD: Parkinson’s disease, PPH: postprandial hypotension, RCT: randomized controlled trial, RF: recurrent falls, RR: relative risk, RRR: relative risk reduction, SACU: Sub-Acute Care Unit, SD: standard deviation, T-LOC: transient loss of consciousness, UF: unexplained falls, VVS: vasovagal syncope.

| **Table 2. Overview of studies on pacemaker intervention** | | | | | | | | | | |
| --- | --- | --- | --- | --- | --- | --- | --- | --- | --- | --- |
| **Author, year** | **Design & data gathering** | **N** | **Mean Age (SD) or median (range)** | **Population** | **Setting** | **CV treatments** | **FU time** | **Outcome (measurement)** | | **Results** |
|  |  |  |  |  |  |  |  | **Fall-related** | **Others** |  |
| Brenner, 2017  [7] | Cohort, prospective | 78 | 75.4 (8.3) | Patients with SND (based on 12-lead or Holter ECG) and class I indication for PPM implantation | Cardiology  department, multicenter | Dual chamber  permanent pacemaker implantation | 12 mon. | Primary: fall incidence.  Secondary: number of falls (with or without injury/requiring medical treatment/fracture).    Fall data:  -before PPM retrospectively collected (patient recall/ history over previous 12-months).  - after PPM prospectively collected. | N/A | 52.6% of patients fell before implantation vs 15.4% after implantation (RRR 71%, p<0.001). 127 falls before implantation vs 13 falls after implantation (RRR 90%, p<0.001). 28% of patients had falls with injury before implantation vs 10% after implantation (p=0.005). 31% had falls requiring medical treatment vs 8% after implantation (p<0.001). 8 % had falls with fracture before implantation vs 0% after implantation (p=0.013) |
| Gillespie, 2012  [13] | Systematic review and meta-analyses | 349 participants (3 trials concerning pacemaker) |  | Fallers CICSH | Community dwelling older people | Cardiac pacemaker insertions |  | Primary outcome: Rate of falls, Number of fallers |  | Cardiac pacing was associated with a statistically significant reduction in rate of falls (RaR 0.73, 95% CI 0.57-0.93) (349 participants, 3 trials).  Not statistically significant associated with risk of falling (RR 1.20, 95% CI 0.92-1.55) (178 participants, 2 trials) or risk of fracture (RR 0.78, 95% CI 0.18-3.39), (171 participants, 1 trial) |
| Krasniqi, 2012  [5] | Cohort, retrospective | 124 | 71.9  (7.9) | Patients with SND (based on Holter ECG) and underwent PM implantation. | Tertiary cardiology  clinic | Pacemaker implantation | Mean 2.3 years | Number of (injured) fallers,  Number of (injurious) falls  Fall data  retrospectively collected from medical records. | N/A | 32.3% of patients fell before PM implantation vs 8.1% after PM implantation (RRR 74.9%, P>0.001).  15.3% of patients had a fall with injury before PM vs 5.6% after PM implantation (RRR63.4%, p=0.014).  Number of falls reduced from 60 to 22. Number of falls with injury from 22 to 7 (RRR 63%, p=0.035 and 67%, p=0.013). |
| Karunaratne, 2002  [10] | Cohort, retrospective | 50 | 71.9  (56.0-88.0) | Patients with CI-CSH, vasodepressor or mixed CSH | Falls clinic | Dual chamber permanent pacemaker | Mean 10 mon. | Falls after pacemaker implantation.  Falls data retrospectively collected. | N/A | 50 patients underwent pacemaker implantation, 28% (n=14) were for CSH.  In the CSH group, 50% (n=7) had unexplained falls prior to pacing. After implantation: 0 had falls. All 7 with falls, completely improved. |
| Bexton, 1997  [9] | Cohort, retrospective | 67 | 73.0 | Patients with CSS | Cardiology unit | Rate-Drop Response (RDR feature of Medtronic Thera pacemaker) | N/A | Falls  retrospectively collected (questionnaires) | Symptoms such as syncope, or dizziness, Injuries events  retrospectively collected | Falls: 21 preimplant vs 8 post implants (p=0.043).  Syncope: 55 preimplant vs 9 post implants (p=<0.001)  Dizziness: 43 preimplant vs 41 postimplant (NS) |
| Crilley, 1997  [4] | Cohort, retrospective | 37 | 79.0 (58.0-91.0) | Patients with recurrent falls, dizziness and syncope and CICSH after 5sec massage of either carotid sinus in supine or erect position; during CSM and/or HUTT). | Geriatric department | Dual chamber  pacemaker | 10 mon.  (1.5-30) | Fall incidence  Retrospectively collected through questionnaire sent to general practitioner. | N/A | 81 % had falls before implantation against 30% after implantation (p<0.0001) |

**Abbreviations:** CI: confidence interval, CI-CSH: cardioinhibitory subtype of carotid sinus hypersensitivity, CSM: carotid sinus massage, CSS: carotid sinus syndrome, CSH: carotid sinus hypersensitivity, CV: cardiovascular, CVD: cardiovascular disease/disorder, Dep.: Department, ECG: electrocardiogram, ED: emergency department, FU: follow-up, HR: hazard ratio, HUTT: head-up tilt test, ILR: implantable loop recorder, N/A: not available/applicable, OH: orthostatic hypotension, OR: odds ratio, PM: pacemaker, PPM: Permanent pacemaker, RCT: randomized controlled trial, RDR: rate-drop response, RR: relative risk, RRR: relative risk reduction, SBP: systolic blood pressure, SD: standard deviation, SND: sinus node dysfunction.

| **Table 3. Overview of studies on orthostatic hypotension-related treatments** | | | | | | | | | | |
| --- | --- | --- | --- | --- | --- | --- | --- | --- | --- | --- |
| **Author, year** | **Design** | **N** | **Mean Age (SD)** | **Population** | **Setting** | **CV treatments** | **FU time** | **Outcome (measurement)** | | **Results** |
|  |  |  |  |  |  |  |  | **Fall-related** | **Others** |  |
| Hauser, 2016  [2] | Multicenter randomized, placebo controlled, double-blind trial | 197 | 72.4 (7.8) | Patients with PD, with neurogenic OH | Parkinson clinic | Droxidopa (100–600 mg, 3× daily) or placebo | 10 weeks | Secondary outcome: number of falls prospectively recorded (daily electronic diary).  Fall-related injuries. | N/A | Droxidopa group (n=92) reported 308 falls and placebo group (n=105) reported 908 falls. Fall rates of 0.4 falls per patient week in droxidopa group vs 1.05 falls per patient week in placebo group (P = 0.014).  Fall related injury: 16.7% in droxidopa group and 26.9% in placebo group. |
| Vilches- moraga, 2012  [22] | Cohort, prospective | 135 | 84.0 | Patients referred for assessment of syncope or unexplained falls with diagnose of neurocardiogenic syncope (OH, VVS, CSH), where non-pharmacological measures failed, or symptoms were severe. | Falls and syncope outpatient unit | Midodrine | 2.7 years | Retrospective: unexplained falls as reason for referral | Prospective: positive response to midodrine therapy (significant reduction, or abolition of symptoms) | 97 (71%) of the patients started on midodrine reported an abolition/significant reduction across all TT/CSM diagnosis. 29% reported no benefit.  49% achieved sustained clinical improvement after initial doses of 2.5mg 3 times a day; only 4 required dosage >7.5mg 3 times per day. Adverse drug reactions were minor (19 subjects), 6 resulted in drug withdrawal. |

**Abbreviations:** CSM: carotid sinus massage, CSH: carotid sinus hypersensitivity, FU: follow-up, N/A: not available/applicable, OH: orthostatic hypotension, PD: Parkinson’s disease, SD: standard deviation, TT: tilt testing, VVS: vasovagal syncope

| **Table 4. Overview of studies on cardiovascular exercise intervention** | | | | | | | | | | |
| --- | --- | --- | --- | --- | --- | --- | --- | --- | --- | --- |
| **Author, year** | **Design** | **N** | **Mean Age (SD)** | **Population** | **Setting** | **CV treatments** | **FU time** | **Outcome (measurement)** | | **Results** |
|  |  |  |  |  |  |  |  | **Fall-related** | **Other** |  |
| Genta, 2017  [11] | Cohort, prospective | 135 | 80.0  (5.0) | Patients after TAVI and sAVR (control) procedure | Cardiac rehabilitation unit | 3-week intensive supervised, tailored aerobic incremental exercise training program | 3 weeks | Secondary: risk of falls with Morse fall scale (MFS) on admission and discharge | Six-min walk test (6MWT) distance, Barthel index, on admission and discharge. Cumulative Illness Rated State-comorbidity Index (CIRS CI) and echocardiography outcomes. | Risk of falls was higher in TAVI than sAVR patients both at admission (MFS: 36+/-22 vs 25 +/-19; p=0.002) and at discharge (MFS: 30 +/-20 vs 20+/-12; p=0.0001). Significant improvement in risk of falls at discharge both in TAVI (MFS difference admission vs discharge: 5.8+/-20; p=0.02) and sAVR patients (MFS difference admission vs discharge: 5+/-15; p=0.005).  TAVI patients were more disabled then sAVR patients both at admission and at discharge. There was a significant improvement in disability and functional capacity in both groups. |

**Abbreviations:** CIRS-CI: cumulative illness rated state-comorbidity index, CV: cardiovascular, FU: follow-up, MFS: morse fall scale, sAVR: surgical valve replacement, SD: standard deviation, TAVI: transcatheter aortic valve implantation, 6MWT:6-min walk test.

| **Table 5. Overview of studies on (orthostatic) blood pressure measurements** | | | | | | | | | | | |
| --- | --- | --- | --- | --- | --- | --- | --- | --- | --- | --- | --- |
| **Author, year** | **Design & data gathering** | **N** | **Mean Age (SD) or median (range)** | **Population** | **Setting** | **CV diagnostics** | **FU time** | **Outcome (measurement)** | | **Results** |  |
|  |  |  |  |  |  |  |  | **Fall related** | **Others** |  |  |
| Hohtari-Kivimäki,  2022 [23] | Cohort, prospective | 561 | 73.1 (65.0-94.0) | Volunteer participants of a multifactorial fall prevention intervention study. Aged ≥ 65 years, good or moderate cognitive abilities, at least 1 fall in previous 12 months, and ability to walk 10 m independently with or without walking aids. | Community dwelling or sheltered housing. | Standard mercury sphygmomanometer.  BP and HR in supine position and at 30 sec and 3min after standing. | 12 & 36 mon. | 1) Number of falls  2) number of falls requiring treatment  Falls data prospectively collected using fall diaries, returned monthly during a 12-month follow-up.  Falls requiring treatment: from health center and hospital registers during 12 and 36 months. | Prevalence of OH at 30 seconds and 3 minutes.  OH was defined as reduction of SBP of ≥20mmHg or DBP of ≥10mmHg within 3 minutes of standing. | Prevalence of OH: 23.4% at 30 sec and 7.3% at 3 min measurement.  30 sec measurements: significantly higher incidence of falls (IRR 1.35, 95% CI 1.10-1.66) and of falls requiring treatment (IRR 2.04, 95% CI1.13-3.66) in OH group compared with non-OH group for 12 months. Adjusted, incidence of falls remained higher in all models; that of falls requiring treatment remained higher only after adjustment for functional balance.  3 min measurement: higher incidence of falls in OH group compared with non-OH group for 12 months (IRR 1.48, 95% CI 1.04-2.02) and remained higher after adjustments for functional balance and for age and functional balance.  During 36-month follow-up OH both at 30 seconds or 3 minutes was not associated with occurrence of falls leading to treatment. |  |
| Juraschek,  2022 [24] | Original RCT used as prospective cohort for current study | 534 | 76.3 (5.3) | Aged ≥ 70 years with low serum 25(OH)D levels (10–29 ng/ml) and elevated fall risk (2 or more falls or 1 injurious fall in past year, fear of falling, difficulty maintaining balance or use of assistive device). | Community dwelling  Participants of the Study to Understand Fall Reduction and Vitamin D in You (STURDY) | Orthostatic BP measurements: seated-to-standing (5 min sitting rest, standing 1 min and 3 additional measurements separated by 30-sec pause) and supine-to-standing protocol (5 min supine rest, standing 3 measurements directly after standing and another 3 measurements timed after 3min standing).  Using Omron HEM907XL. | Median 1.7 years | Number of falls  Prospectively collected using monthly fall calendars, scheduled clinic visits and telephone calls and ad hoc telephone contacts (participants called the clinic if they fell). | -Prevalence of OH  -Orthostatic symptoms  OH was defined as a drop in systolic or diastolic BP of at least 20 or 10 mmHg. | OH was detected in 2.1% (SE 0.5) of seated vs 15% (SE 1.4) of supine assessments (P<0.001).  Supine OH was associated with self-reported fainting, blacking out, seeing spots and room spinning in the prior month (P-value < 0.03); sitting OH was not associated with any symptoms (P-value ≥ 0.40).  Supine and seated OH were not associated with falls (HR: 1.55 [0.95, 2.52] vs 0.69 [0.30, 1.58]). Supine systolic OH was associated with higher fall risk (HR: 1.77 [1.02, 3.05]).  All models were adjusted. |  |
| Mol, 2022 [25] | Cohort, cross-sectional | 635 | 81.1 (6.9) | Patients referred to a geriatric outpatient clinic undergoing a comprehensive geriatric assessment. | Geriatric outpatient clinic | Orthostatic BP measurement using a sphygmomanometer during supine rest and 1 and 3 min after standing up | N/A | Number of falls  Retrospective: using a questionnaire asking for the number of falls in the past year. | BP recovery (BP at 3 min minus BP at 1 min after standing)  Physical performance: using TUG and SPPB. | BP recovery was not associated with number of falls or physical performance, neither in the entire cohort nor in subpopulations with or without OH. Adjusted models. |  |
| Welmer, 2020  [26] | Cohort, prospective | 3055 | 73.7 (10.6) | Participants of the Swedish National study on Aging and Care, aged ≥ 60 years | Community-dwelling | BP measurements by mercury sphygmomanometer. Sitting at least 5 min  followed by lying at least 5-min, and secondly 1 min after standing position. | 10 years | Retrospective:  Injurious falls leading to inpatient or outpatient care during 3 and 10 years of follow-up were identified via patient registers. | BP categorized as <130, 130-139 (reference), 140-159 and ≥ 160 for SBP and <70, 70-89 (reference), ≥90 mmHg for DBP.  Mean arterial pressure (MAP): (SBP x 1/3) + (DBP x 2/3)  Pulse pressure (PP): difference between SBP and DBP.  OH: decrease in SBP of at least 20mmHg or DBP of at least 10mmHg after assuming standing position.  Physical function: tests of balance, chair stands, walking speed | During 10-year follow-up: without functional impairment, adjusted HR of injurious falls were 1.77 (95% CI 1.02-3.07) for having SBP <130mmHg, 1.73 (95% CI 1.05-2.83) for SBP≥160mmHg (vs 130-139) and 1.46 (95% CI 1.05-2.02) for having higher tertile of PP (vs lower tertile).  During 3-year follow-up: with functional impairment, adjusted HR for injurious falls was 1.91 (95% CI 1.17-3.13) for SBP <130mmHg and 0.74 (95% CI 0.59-0.94) for having higher tertile of PP (vs lower tertile).  No significant association between BP components and injurious falls in people without functional impairment during 3-year follow-up or in people with functional impairment during 10-year follow-up. |  |
| Mol, 2019  [27] | Systematic review and meta-analysis | SR: 63 studies; MA:50 studies (N=49164) | N/A | Inclusion criteria: English, mean/median age ≥65 years, blood pressure measurement before and after postural change, assessment of the association of OH with falls. | Community dwelling, geriatric outpatients and inpatients, nursing home residents, patients with PD/specific other diseases. | Subgroup analyses for blood pressure measurement method: Continuously blood pressure measurement vs intermittently blood pressure measurements. | N/A | Assessment of falls | BP measurements before and after postural change. Assessment of the association of OH with falls.  Blood pressure measurement method. | OH was positively associated with falls (OR 1.73; 95% CI 1.50-1.99); independent of population, design, quality, OH definition and measurement method.  Association between OH and falls was strongest in the subgroup of studies using continuous blood pressure measurements (OR 2.35, 95% CI 1.76-3.13 vs 1.66, 95% CI 1.43-1.93).  Intermittent BP measurement method: (OR 1.66, 95% CI 1.43-1.93).  Unadjusted models. |  |
| Van der Velde, 2007  [28] | Case-control, cross sectional | 217 | Fallers: 78.8 (5.7)  Non fallers: 75.0 (6.0) | Patients referred to the outpatient clinic, aged ≥ 65 years | Outpatient clinic | Sphygmomanometer BP measurements, supine after 10min rest and standing at 1,2 and 3 min.  Continuous-finger-blood pressure measurements (Finapres) at 10 min supine rest and 70 degrees tilted. | N/A | Retrospective: positive fall history (at least one fall within previous year), falls data. | Sphygmomanometer BP measurement: lowest BP.  Continuous blood pressure measurement: beat-to-beat values and 1, 5, 10, 15, 20 and 30-s averages.  OH was defined as a 20mmHg fall in SBP or a 10mmHg fall in DBP. | The best association with falls history was found for the 5-sec average.  Falls and OH did not correlate by sphygmomanometer.  OR of a fall according to OH using the 5-sec average was 2.54 (95% CI 1.37-4.71). Unadjusted models. |  |
| Aronow,  1994  [29] | Cohort, prospective | 499 | 80.0  (9.0) | Residents aged ≥62 years able to assume the sitting position | Long-term healthcare facility | BP measurements (Calibrated mercury sphygmomanometer) before lunch, and at 15, 30, 45, 60, 75, 120 min after lunch | N/A | Retrospective: data on falls and syncope were collected over preceding 6 months. | Pre- and postprandial BP, max decrease of BP | Residents with a history of syncope/falls have a significantly greater maximal decrease in postprandial SBP than residents without a history of syncope/falls (21 +/- 5 mm Hg in residents with falls in the preceding 6 months and 13 +/- 4 mm Hg in residents without falls (P < 0.0001)).  Multivariate analysis: residents with a history of syncope/falls have a significantly greater maximal decrease in postprandial SBP than residents without a history of syncope or falls after controlling the effects of medications (P < 0.0001). |  |
| Aronow, 1997  [30] | Cohort, prospective | 499 | 80.0  (9.0) | Residents aged ≥62 years able to assume the sitting position. | Long-term healthcare facility | BP measurements (Calibrated mercury sphygmomanometer) before lunch, and at 15, 30, 45, 60, 75, 120 min after lunch | 29 mon (mean) | Incidence of falls.  Prospective: data on falls and episodes of syncope collected on forms. | Maximum decrease in postprandial systolic BP  Incidence of new coronary events, new stroke and total mortality. | Falls had occurred in 199 persons (40%), syncope in 73 (14%), new coronay events 139 (28%), new stroke 61 (12%) and total mortality 199 (40%).  The mean max decrease in postprandial SBP was 20+/- 5 mm Hg in persons with falls and 12 +/-4 mmHg in persons without falls (P < 0.001). 23+/-5 mm Hg in persons with syncope and 14+/- 5 mm Hg in persons without syncope (P < 0.001). Max decrease in postprandial systolic BP was independent risk factor for falls (risk ratio 1.19, 95% CI 1.15-1.23), syncope, coronary events, stroke and total mortality. Adjusted models. |  |
| Donoghue,  2021  [31] | Cohort, prospective, cross-sectional | 1500 | 71.1 (5.2) | Age ≥65 years, MMSE score ≥18 and no PD or cognitive impairment | Community, population-based  TILDA cohort. | Continuous beat-to-beat BP measurements using  digital photoplethysmography (Finometer device) during active stand procedure with 10 min supine rest and 2 min standing. | Mean follow-up time 4.1 years; (range 2.8-5.7 years). | A number of dichotomous falls variables (recurrent falls, injurious falls, unexplained falls, and syncope)  Retrospective from self-reported falls interviews on the past year/since the last interview. | OH, OH at 40seconds (OH40) and OH sustained over the second minute (sustained OH)  Seated & supine hypertension (SBP >140mmHg/ DBP>90mHg)  OH was defined as drop in SBP ≥20 mmHg and/or ≥10 mmHg in DBP within 3 minutes of standing.  Impaired stabilization (OH40) was defined as a drop in SBP ≥20 mmHg and/or ≥10 mmHg in DBP at 40 seconds. | OH40 was associated with recurrent falls (RR = 1.35, 95% CI = 1.08-1.68); injurious falls (RR = 1.44, 95% CI = 1.16-1.78) and unexplained falls (RR = 1.57, 95% CI = 1.16-2.13). Sustained OH was also associated with injurious (RR = 1.55, 95% CI = 1.18,2.05) and unexplained falls (RR = 1.63, 95% CI = 1.06,2.50), but not recurrent falls. Adjusted models.  OH, and co-existing hypertension was associated with all fall outcomes, with stronger effect sizes with seated hypertension. |  |
| Moloney,  2021  [32] | Cohort, prospective, cross sectional | 4899 | 61 (8.8) | Aged ≥50 years | Community, population-based  TILDA cohort | Continuous BP monitoring (Finometer device) during active stand test after 10min of supine rest.  BP response data up to 120 sec post-stand, at 10 sec interval. | N/A | Retrospective: history of falls (previous 12 months) and black out in the past 12 months. | 8 AS patterns based on three binary SBP deficits: SBP drop ≥40mmHg within 10sec post-stand (immediate drop: yes/no); failure to return to within 20mmHg of supine level at 40sec after standing (stabilization failure: yes/no); drop ≥20mmHg at any time between >40 and 120sec post-stand (late deficit: yes/no).  Orthostatic intolerance: self-reported dizziness, light-headedness or unsteadiness during AS. | Largest group was the one with no deficits (68%), followed by immediate deficit only (13%), all three deficits (6%) and late deficit only (5%). Older age was associated with stabilization deficit.  Late deficits were seen in groups with higher proportion of beta-blockers and psychotropics. Groups with an immediate deficit had higher risk of Orthostatic intolerance.  Significant association between falls history and the three-deficit group (OR 1.54, 95% CI 1.15-2.07). Adjusted models. |  |
| Mol, 2020  [33] | Cohort, prospective, cross-sectional | 168 | 81.4  (7) | Older adults referred to geriatric outpatient clinic for cognitive or mobility problems | Geriatric outpatient clinic | Continuous BP measurements using finger photo plethysmography (Nexfin) during a standardized AS test with 5 min of supine rest and 3 min of standing. | N/A | Number of falls; self-reported in the past year.  Retrospective collected. | BP drop rate: the largest amplitude of the negative peak in the first derivative of BP.  BP drop magnitude: the magnitude of the largest decline in BP compared with the baseline.  Orthostatic HR increase: the max HR within 15sec after baseline.  Baroreflex sensitivity: HR max increase divided by SBP drop magnitude 0-15sec.  Frailty according to Fried criteria. | SBP drop rate was associated with number of falls (β 1.09; 95% CI, 0.19–1.20; P=0.018) and with frailty (β 0.30; 95% CI, 0.11–0.49; P=0.003).  DBP drop magnitude was most strongly associated with frailty (β 0.37; 95% CI, 0.15–0.60; P<0.001).  Baroreflex sensitivity was not associated with number of falls or frailty.  Adjusted models. |  |
| Finucane,  2017  [34] | Cohort, prospective, cross-sectional | 4127 | 61.5 (8.2) | Aged ≥50 years | Community, population-based  TILDA cohort | Continuous BP measurements (Finometer device) during AS after 10 min supine rest, remained standing for 2 min. | 2 years | Retrospective  Fall incidents: have you fallen since last interview?  Number of falls: How many times have you fallen?  Unexplained falls (UF): Were any of these falls non accidental (with no apparent or obvious reason)?  Injurious falls (IF): did you injure yourself seriously enough to need medical treatment?  (Same questions for syncope) | Initial OH (iOH): minimum BP on standing within 15 sec that exceeds thresholds for iOH (drop of SBP ≥40mmHg and/or DBP ≥20mmHg).  OH at 40 seconds (OH40): failure to return to within SBP ≥20mmHg and/or DBP≥10mmHg of supine levels at 40 sec after standing.  OH: sustained failure of SBP or DBP to stabilize to within 20mmHg SBP or 10mmHg DBP of supine levels from 60-110 sec after active standing. | OH associated with all cause falls (IRR 1.40 95% 1.01-1.96), UF (RR 1.81; 1.06-3.09) and IF (RR 1.58; 1.12-2.24).  OH40 was associated with increased relative risk of UF (RR: 1.52 95% CI 1.03-2.26). iOH was not associated with any outcome. Adjusted models. |  |
| Goh,  2017  [35] | Cohort, prospective, cross sectional | 1218 | 69.0  (64.0-75.0) | Participants, Aged ≥55 years, from the Malaysian Elders Longitudinal Research study (MELoR) | Community, population based. | Continuous noninvasive beat-to-beat BP measurements (Task Force, CN System) during AS test (10 min supine rest and 3 min AS) | N/A | Retrospective: falls in previous 12 months. | Time-domain and frequency-domain blood pressure variability (BPV)  Standard deviation (SD) and root mean square of real variability (RMSRV) for time-domain BPV.  Fast-Fourier transform low frequency (LF), high frequency (HF), total power spectral density (PSD), and LF:HF ratio for frequency domain BPV. | Comparisons between individuals with at least one fall (n=256;21%) and non-fallers. Standing SBPV was significantly lower in fallers (SBPV-SD, P=.016; SBPV-RMSRV, P=.033; SBPV-LF, P=.003; SBPV-total PSD, P=.012).  Non-fallers had significantly higher supine to standing ratio (SSR) for SBPV-SD, SBPV-RMSRV, SBPV-total PSB (P=.017, P=.013, P=.009).  In multivariate analyses, standing BPV remained significantly lower in fallers (adjusting for age, sex, diabetes, frailty walk and supine systolic BP).  Reduction in frequency-domain SSR among fallers was attenuated by supine systolic BP, TUG and frailty walk. |  |
| Goh, 2016  [36] | Case-control, prospective | 50 | Fallers: 76±8; non-fallers: 76±5 | Fallers (n=25), aged ≥65years; two or more falls or one injurious fall in past year  Non-fallers (n=25) from MEloR research database. | Fallers recruited from ED, primary care clinics, geriatric outpatients. | Continuous noninvasive beat-to-beat BP measurements (Task Force) during AS (10 min supine rest and 3min AS) | N/A | Retrospective: two or more falls or one injurious fall in previous year were included. | Systolic blood pressure variability (BPV) and diastolic BPV were assessed using 5 indices: standard deviation (SD), standard deviation of most stable continuous 120 beats (staSD), average real variability (ARV), root mean square of real variability (RMSRV) and standard deviation of real variability (SDRV). | Standing SBPV was significantly higher than supine using 4 indices in both groups.  Standing to supine BPV-ratio (SSR) was then computed for each subject (staSD, ARV, RMSRV, SDRV). Standing-to-supine ratio for SBPV was significantly higher among fallers compared to non-fallers using RMSVR and SDRV (p=0.034 and p=0.025); Unadjusted models  3 indices (ARV, RMSRV, SDRV) of SSR SBPV provided accuracies of 61.6%, 61.2% and 60% for the prediction of falls which is comparable with TUG (64.4%). |  |
| Jonas,  2018  [37] | Cohort, retrospective | 1032 | Fall injury: 78.2 (4.4)  No fall injury: 76.3 (5.1) | Community dwelling older patients (>70 years) with hypertension referred for 24-h ABPM | Outpatient clinic | 24-h ABPM | 1 year | Prospective: fall injuries within one-year post-ABPM. From patient records (with diagnosis of falls based on ICD-9 diagnosis). | Clinical characteristics, 24-h ABPM patterns and intensification of hypertensive therapy following 24-h ABPM in patients with and without fall injury. | 55 (5.3%) had a fall injury in the year following ABPM. Patients with fall injury had significantly lower 24-h DBP (67.3 +/- 7.6 vs 70.7 +/- 8.8; P<0.005) and a higher pulse pressure (74.7 +/-14.3 vs 68.3 +/-13.7 mmHg; p>0.005), both awake and sleep. After adjusting for age, gender, DM, previous falls: lower DBP and increased pulse-pressure were independent predictors for fall injuries.  Intensification of antihypertensive treatment following 24-h ABPM was not associated with increased rate of fall injury. |  |
| Le Couteur, 2003  [38] | Observational series, cross-sectional | 179 | 83.2 (7.0) | Semi-independent living older adults | 8 Residential care facilities | ABPM (oscillometric cuff method).  BP and HR measurements 1) lying, fasted, 2) after sitting 15 min before breakfast, 3) sitting, 60 min after breakfast, 4) after standing for 1 min, 5) after standing 3 min, 6) after 10 min walk, 60 min after breakfast. | N/A | Retrospective: falls data available from diary records over 12 months. | PPH, defined as ≥20mmHg fall in systolic BP.  Absolute level of SBP after the meal. | PPH and low absolute SBP (<115mmHg) were common after meals and exacerbated by standing. A SBP fall of 20 mm Hg or more after meals was not associated with any increase in the overall risk of falling in the last year (OR = 1.0, CI 0.6–1.9) or of recurrent falls in the last year (OR = 0.9, CI 0.4–1.9). Those in whom seated SBP declined to 115 mm Hg or less after a meal had increased risk of falling (OR = 3.7, CI 1.3–11.1, p = 0.013). Unadjusted models.  The incidence of falls was increased only in the 10% of subjects with low postprandial SBP. |  |
| Puisieux,  2000  [39] | Case-control, Cross-sectional | 152 | Fallers: 80.9 (8.5) syncope: 81.4 (8.2). Controls: 78.5 (7.2) | Older adults (≥60 years) admitted for syncope or falls (n=120).  Controls: inpatients without history of falls or LOC (n=43) | Geriatric short stay department | 24h-ABPM; readings every 15 min during the day and every 30 min at night. Meals at set times. | N/A | Admitted for any fall. | Postprandial SBP decline pre-prandial SBP – postprandial SBP. PPH was defined by change in SBP ≥20mmHg. | SBP change was slightly greater among the patients with syncope or falls than among controls (respectively, 4.5 +/-5.7, and 3.8 +/- 7.5 vs 1.8 +/- 6.6 in the control group; p=.015).  Number of patients with PPH was significantly higher in the syncope/fall group than in the control group (23% vs 9%; p= .03). Decline in SBP was greater after breakfast.  All patients experienced at least one (after one of the three meals) decline in SBP. |  |

**Abbreviations:** ABPM: ambulatory blood pressure monitoring, AS: active stand, ARV: average real variability, BP: blood pressure, BPV: blood pressure variability, CI: confidence interval, CV: cardiovascular, CVD: cardiovascular disease/disorder, DBP: diastolic blood pressure, ECG: electrocardiogram, ED: emergency department, FU: follow-up, HR: hazard ratio, HR: Heart rate, IF: injurious falls, IRR: incidence rate ratio, iOH: Initial OH, N/A: not available/applicable, LOC: transient loss of consciousness, OH: orthostatic hypotension, OI: orthostatic intolerance, OR: odds ratio, PD: Parkinson disease, PPH: postprandial hypotension, RCT: randomized controlled trial, RMSRV: root mean square of real variability, RR: relative risk, SBP: systolic blood pressure, SBPV: systolic blood pressure variability, SD: standard deviation, SDRV: standard deviation of real variability, SPPB: Short Physical Performance Battery, SSR: standing to supine ratio, mon.: months, TUG: timed-up and go, UF: unexplained falls.

| **Table 6. Overview of studies on arterial stiffness measurements** | | | | | | | | | | |
| --- | --- | --- | --- | --- | --- | --- | --- | --- | --- | --- |
| **Author, year** | **Design & data gathering** | **N** | **Mean Age (SD) or median (range)** | **Population** | **Setting** | **CV diagnostics** | **FU time** | **Outcome (measurement)** | | **Results** |
|  |  |  |  |  |  |  |  | **Fall-related** | **Others** |  |
| Turusheva,  2020  [40] | Cohort, cross-sectional | 163 | 70.9 (6.5) | Community-dwelling older adults aged ≥60 years | Community dwelling | Cardio-Ankle Vascular Index (CAVI); ECG electrodes on wrists, microphone for phonocardiography on sternum and four BP cuffs around the limbs. Upper arm and ankle pulse waves, and BP are measured. CAVI is disregarded if the ankle–brachial index is less than 0.9 | N/A | Retrospective: falls or self-reported fall-related injuries during last year. | High CAVI: CAVI above the predicted value in accordance with the proposed formula.  Finding of OH (decrease in SBP ≥ 20 mm and/or decrease in DBP ≥ 10 mm within 3 minutes after standing. | CAVI above predicted value was associated with fall-related injuries (OR 3.52, 95% CI 1.03-12.04), adjusted.  OH in 4.9% (n=8) participants, but it was not associated with high CAVI or fall-related injuries.  Women with high CAVI had a more pronounced decline in SBP in minute 2 when performing the orthostatic test compared with those with low CAVI. |
| Wong,  2014  [41] | Cohort, prospective | 473 | 79.8 (4.4) | Participants from Wave 2 of the Sydney Memory and Ageing Study (MAS). Cardiovascular and fall risk assessments were performed in a subsample. | Community dwelling | Carotid-femoral pulse wave velocity (PWV) measured in supine position after lying for 10 minutes using a semi-automated pulse wave analysis system. BP measurement seated (electronic sphygmomanometer), supine and tilted BP using tilt-table (70 degrees tilted). | 12 mon. | Prospective: falls frequency during follow-up using monthly falls diaries. | PWV (as measure of arterial stiffness), BP and diagnosis of OH.  OH was defined as a reduction of 20 mmHg or more in SBP or 10 mmHg or more in DBP within 3 minutes of tilting. | Participants with high PWV were more likely to have higher seated SBP and HR, unsatisfactory control of BP and lower physical activity levels. Of the 473 participants available for follow-up, 212 (44.8%) reported one or more falls.  In modified Poisson regression analyses, high PWV was a risk factor for falls (relative risk = 1.37, 95% CI = 1.06–1.78), adjusted.  Fallers and non-fallers did not differ with respect to presence of OH. |

**Abbreviations:** BP: blood pressure, CAVI: Cardio-Ankle Vascular Index, CI: confidence interval, CV: cardiovascular, CVD: cardiovascular disease/disorder, DBP: diastolic blood pressure, ECG: electrocardiogram, FU: follow-up, HR: heart rate, N/A: not available/applicable, OH: orthostatic hypotension, OR: odds ratio, PWV: pulse wave velocity, SBP: systolic blood pressure, SD: standard deviation.

| **Table 7. Overview of studies focusing on heart rhythm assessments** | | | | | | | | | | |
| --- | --- | --- | --- | --- | --- | --- | --- | --- | --- | --- |
| **Author, year** | **Design & data gathering** | **N** | **Mean Age (SD) or median (range)** | **Population** | **Setting** | **CV diagnostics** | **FU time** | **Outcome (measurement)** | | **Results** |
|  |  |  |  |  |  |  |  | **Fall-related** | **Others** |  |
| Isik,  2012  [42] | Case-control, prospective | 64 | Fallers: 74.7 (6.1)  Control: 73.5 (3.8) | Patients ≥65 years who had falls in the last 12 months (n=33)  Control: non-fallers (n=31) | Geriatric outpatient clinic | 24-h Holter monitoring | N/A | Retrospective: falls history in previous 12 months. | HRV, QT dynamicity and HRT parameters  LV end-diastolic volume, end-systolic volume, ejection fraction. | There were 33 fallers and 31 non-fallers.  No significant differences in HRV and QT dynamicity parameters between groups. Both HRT onset and turbulence slope were significantly different between the groups. |
| Davison, 2005  [43] | Case-control, cross-sectional | 228 | Fallers: 76.8 (6.2)  Non-fallers: 75.0 (5.0) | Older adults aged ≥65years presented to the accident and ED with a fall or fall-related injuries and had an additional fall in preceding 12 months (n=128).  Matched controls (n=100) without falls recruited from GP population. | ED | 24-hour ambulatory electrocardiography | N/A | Retrospective using diary recordings (time, circumstances and symptoms associated with falls during recording episode). | Electrocardiographic abnormalities categorized Major: ventricular arrhythmia, pauses, <30 bpm., Mobitz II, complete heart block)  Minor: multiple ectopics, paroxysmal atrial arrhythmia, other bradyarrhythmias | 49% (63) of recordings in fallers and 41% (41) of recordings in controls were abnormal (at least one ECG abnormality; non-significant difference)  The prevalence of abnormalities did not differ between fallers and controls. Mild symptoms (included palpitations) in 10% fallers vs 13% controls (non-significant difference). One patient fell during monitoring with no associated rhythm abnormality. |
| Rosado, 1989  [44] | Case-control, cross-sectional | 78 | 86.0 | Participants with a fall (n=51) within past seven days and ability to ambulate independently  without an assistive device.  Non-faller control subjects (n=27) were obtained by random selection of residents who did not fall in preceding 12 months. | Residential care facility | Ambulatory cardiac (Holter) monitoring (AHM) within seven days of the fall and for controls shortly after their selection. | 12 mon. | Retrospective: any fall in past seven days. | Prevalence of  ventricular arrhythmias | 78 AHMs from 51 fallers and 27 controls. Prevalence of ventricular arrhythmias was 82% in each group. All patients had supraventricular arrhythmias.  There was no difference in the severity of arrhythmias between fallers and non-fallers. No symptoms were reported during the Holter monitoring. |
| Gordon, 1982  [45] | Cohort, retrospective | 59 | 82.0 | Residents that experienced dizziness, palpitations, syncope, chest pain, or falls that were not explained by physical examination and standard ECG. | Geriatric residential care setting | 24-hour AHM | N/A | Retrospective: any falls unexplained | Presence of arrhythmias,  symptomatic improvement after antiarrhythmic therapy. | Falls occurred in 37 subjects (62.7%).  12 (of 37) 'fallers' cardiac arrhythmias played a contributing role, in 2 these have been detected in previous ECGs.  22 non-fallers, 4 had abnormal AHM records, 2 arrhythmias have bene detected in previous ECGs. 4 of the 16 subjects with diagnostic AHM record had matching ECGs.  All 16 patients had good response to treatment (disappearance of arrhythmia). |
| Midttun, 2011  [46] | Observational series, retrospective | 207 | 82.8 (58.0-95.0) | Patients referred to the falls clinic or hospitalized due to a fall (during stay in the hospital or as outdoor patient). Patients experienced more than one unexplained fall, severe balance problems, syncope or vertigo. | Geriatric department, falls clinic | event recorder (R-test) | 5-7 days | Retrospective: unexplained fall as indication for implantation event recorder. | Presence of arrhythmias | No arrhythmias in 136 patients.  71 (34%) patients needed regulation of their heart rhythm (by reducing/increasing the existing treatment or supplementing a new drug). 16.4% needed consultation by a cardiologist and 2% of them needed pacemaker. |
| Bhangu, 2016  [47] | Observational series, prospective | 70 | 70.0 (10.0) | Recurrent faller >50 years with two or more unexplained falls.  A fall was unexplained when there was no cause apparent, either intrinsic or extrinsic. | ED | Implantable loop recorder (ILR) | 9 mon. (mean) | After intervention: fall data prospective collected through weekly symptom diaries with regular telephone prompting. | Primary: Cardiac arrhythmia associated with fall or syncope after ILR implantation.  Secondary: detection of cardiac arrhythmia independent of falls/syncope after ILR implantation.  Or fall/syncope without associated arrhythmia | 70 patients received an ILR. In 50 (71.4%) a cardiac arrhythmia was detected by ILR, within 47.3 days (mean). In 14 (20%), falls were attributable to a modifiable cardiac arrhythmia; 14% (n=10) received a cardiac pacemaker, 6% (n=4) received treatment for supraventricular tachycardia.  Patients where a cardiac arrhythmia was detected were more likely to experience a further fall (p=0.0012). |
| Maggi, 2014  [48] | Observational series, retrospective | 58 | 71.0  (17.0) | Patients with initial evaluation for T-LOC of uncertain cause (e.g. unexplained fall) by other specialists (e.g., geriatrists) with suspicion of arrhythmic syncope | Syncope unit | ILR | 20±13 mon. | Retrospective: unexplained falls as indication for ILR. | Diagnostic value of ILR in distinguishing syncope from non-syncopal forms of T-LOC. | During follow-up 33 (57%) patients a spontaneous event was documented by ILR. Diagnosis of arrhythmic syncope was established by ILR in 15 (26%) patients. In 7 of 29 patients (24%) with unexplained falls an arrhythmic syncope was diagnosed by ILR. In 9 patients (16%) there was a fall without detected arrhythmia.  In 13 patients (43%) ILR was unable to document any syncopal episode. |
| Martinez, 2014  [49] | Observational series, retrospective | 13 | 78.0  (73.0-85.0) | Older adults with recurrent unexplained falls (2 or more in previous 2 years), suspected syncope and/or ECG abnormalities. | Cardiology department | ILR | 24 mon. (mean) | Retrospective: unexplained falls as indication for ILR | Symptom-rhythm (arrhythmia) correlation | Average fall rate was 3.3. 3 patients have not presented with a new fall after implantation.  An arrhythmogenic diagnosis was obtained in 5 patients (5/13): bradycardia was identified in 4, tachycardia in 1. The symptoms did not coincide with documented arrhythmia in rest of patients. |
| Armstrong, 2003  [50] | Observational series, retrospective | 15 | 73.0 (61.0–89.0) | Patients ≥ 60 years who had  implantation of reveal for investigation of syncope/unexplained falls | Fall and syncope clinic / CV investigation unit | Patient activated ILR (Reveal) | 18 mon. | Retrospective: unexplained falls as indication for ILR. | Symptom-rhythm correlation | Presenting symptoms were unexplained falls in 3, syncope in 6 and 6 both syncope and falls. Seven (7/15) have activated the device successfully 4 months after implantation. 4 out of 7 had a significant rhythm disturbance (identified bradycardia in 3 and ventricular tachycardia in 1). 3 subjects with bradycardias had dual chamber pacemaker implantation. All subjects with arrhythmias had normal resting 12-lead ECG. |

**Abbreviations:** AHM: ambulatory Holter monitoring, BP: blood pressure, CV: cardiovascular, CVD: cardiovascular disease/disorder, ECG: electrocardiogram, ED: emergency department, FU: follow-up, HR: hazard ratio, HRT: Heart rate turbulence , HRV: heart rate variability, ILR: implantable loop recorder, mon.: months, N/A: not available/applicable, OH: orthostatic hypotension, OR: odds ratio, RR: relative risk, SD: standard deviation, T-LOC: transient loss of consciousness.

| **Table 8. Overview of studies focusing on carotid sinus massage and tilt table testing** | | | | | | | | | | |
| --- | --- | --- | --- | --- | --- | --- | --- | --- | --- | --- |
| **Author, year** | **Design & data gathering** | **N** | **Mean Age (SD) or median (range)** | **Population** | **Setting** | **CV diagnostics** | **FU time** | **Outcome (measurement)** | | **Results** |
|  |  |  |  |  |  |  |  | **Fall-related** | **Others** |  |
| Jansen,  2016  [51] | Systematic review | 86 studies | 50.0-88.0 years | Aged 50 years and older, studies addressing cardiovascular risk factors and falls. | Community dwelling and institutionalized | Selected topics: CSM, HUT table testing | N/A | Falls as an outcome measure. | Diagnosis or assessment of cardiovascular abnormalities | 21 studies on CSH. Reported prevalence between 8% and 73%. 18 studies performed CSM upright and supine (70 degrees); 2 only supine. All defined CSH as asystole greater than 3 sec on ECG or vasodepressor drop of 50mmHg in SBP. 5 studies used symptom reproduction during CSM to differentiate CSS from CSH.  10 studies on VVS. VVS was more common in fallers, prevalence reported between 3% and 46%. All used HUT test as measurement method. |
| Sachpekidis  2009  [52] | Case-control, prospective | 51 | Case: 77.2 (7.9)  Control: 78.1 (6.4) | Hip fracture patients  Matched Control: outpatients, matched by age, sex, cognitive function, comorbid diseases and chronic medical medications. | Hospital | CSM supine HUT 70°, with TTT under continuous BP measurements (intravenous line in radial artery) and continuously ECG recording. | N/A | Retrospectively collected; falls as indication for participation.  Patients were categorized into two groups: group A: accidental falls. Group B: unexplained falls | Outcomes of CSM: CSH | 18 patients (35.3%) had unexplained falls and 33 (64.7%) had accidental falls. 12 in group B (66.7%) had positive response to CSM and 6 in group A (18.2%) (p<0.001). 9 controls (17.6%) also demonstrated CSH.  Patients in Group B were older and were more likely to have a history of unexplained falls or syncope in the past than individuals in group A. Vasodepressor/mixed forms accounted for the majority of CSH responses in Group B (75%). When compared with the control group, CSH was still more common in Group B, but not in Group A. |
| Kerr,  2006  [53] | Observational series, cross sectional | 272 | 71.0 (65.0-92.0) | Participants aged ≥65 years randomly sampled. | General practice register, community dwelling | CSM, supine and upright (tilted) with continuous heart rate and phasic BP monitoring. | N/A | Retrospective: history of falls (no) used for subgroup analyses. | Diagnose of (CI or vasodepressor) CSH | CSH was present in 107 individuals (39%) of the study group. 24% had asystole of 3s or greater during CSM; and 16% had symptoms (including syncope) during CSM. Age and male sex were the only predictors of CSH. Syncope, falls or dizziness in previous 12 months did not predict CSH.  In 80 previously asymptomatic individuals (e.g. no history of falls) CSH was present in 28 (35%) and accompanied by symptoms in 10.  For those with any history of falls, syncope or dizziness and defining a positive response as CSH, the sensitivity of CSM was 41%, specificity 64%, positive predictive value 73%, negative predictive value 32%. |
| Richardson, 2002  [54] | Original RCT used as prospective cohort for current study | 64 | 71.8 (10.8) | Patients aged ≥50 years with CICSH and unexplained or recurrent falls randomized to the control group (no pacemaker insertion) | Syncope facility | CSM (right and left sided, supine and upright at 70 degrees head-up tilt) on four occasions (two before randomization, at 6 months and 1 year following randomization) | 1 year | Retrospective: unexplained/recurrent falls as indication for CSM | Finding of CICSH (diagnosed when CSM produced asystolic response of 3 or more seconds) | CICSH was demonstrated on 82% of occasions. On 75% of these CICSH was demonstrated on right CSM and 77% whilst subject was supine. Before randomization and at 6 months and 1 year, 91%, 67% and 70% of subjects had reproducible CICSH. 50% had CICSH on all four occasions. 17% had consistent response on same side in same position. |
| Schoon,  2013  [55] | Case-control, cross-sectional | 105 | 78.8 (7.0) | Older adults >65 years referred for falls, dizziness and/or syncope (n=105) and 25 community dwelling healthy older subjects. | Geriatric falls clinic | BP measurements using continuous BP measurements (Finapres), head turning test (HTT) after 10 min active standing (until stable BP for 5 min). 3 different head movements were performed during 10-15 seconds: rotation to the right, to the left and hyperextension. CSM to assess CSH using TT, AS test to assess OH, meal test to assess PPH. | N/A | Retrospective: falls as indication for evaluation. | Diagnosis of head turning induced hypotension (HTIH): drop of at least 20mmHg during head movement.  Diagnoses of CSH, OH or PPH using consensus definitions. | Head turning led to hypotension in 39% of patients (mean SBP drop 36mmHg) and in 44% of healthy elderly, irrespective of direction. CSH was associated with HTIH (OR 3.5, 95% CI 1.48-8.35).  Prevalence of OH and PPH in the total group was 48% and 53%. Diagnosis of OH was 42.2% in HTT negative group and 56.1% HTT positive group (non-significant). Diagnosis of PPH was 54.7% in HTT negative group vs 51.2% in HTT positive group (non-significant) |

**Abbreviations:** AS: active standing, BP: blood pressure, CICSH: cardioinhibitory subtype of carotid sinus hypersensitivity , CSM: carotid sinus massage, CSH: carotid sinus hypersensitivity, CV: cardiovascular, CVD: cardiovascular disease/disorder, ECG: electrocardiogram, FU: follow-up, HTIH: head turning induced hypotension, HTT: head turning test, mon.: months, HUT: head up title, N/A: not available/applicable , OH: orthostatic hypotension, PPH: postprandial hypotension, TT: tilt testing, TTT: tilt-table testing, VVS: vasovagal syncope.

**References**

1. Sterne, J.A.C., et al., *RoB 2: a revised tool for assessing risk of bias in randomised trials.* BMJ, 2019. **366**: p. l4898.

2. Hauser, R.A., et al., *Droxidopa and Reduced Falls in a Trial of Parkinson Disease Patients With Neurogenic Orthostatic Hypotension.* Clinical Neuropharmacology, 2016. **39**(5): p. 220-226.

3. Sterne, J.A., et al., *ROBINS-I: a tool for assessing risk of bias in non-randomised studies of interventions.* BMJ, 2016. **355**: p. i4919.

4. Crilley, J.G., et al., *Permanent cardiac pacing in elderly patients with recurrent falls, dizziness and syncope, and a hypersensitive cardioinhibitory reflex.* Postgraduate Medical Journal, 1997. **73**(861): p. 415-418.

5. Krasniqi, N., et al., *Falls and fractures in the elderly with sinus node disease: the impact of pacemaker implantation.* Cardiology Research & Practice, 2012. **2012**: p. 498102.

6. Jansen, S., et al., *Effectiveness of a Cardiovascular Evaluation and Intervention in Older Fallers: A Pilot Study.* Journal of the American Geriatrics Society, 2015. **63**(10): p. 2192-2193.

7. Brenner, R., et al., *Reduction of falls and fractures after permanent pacemaker implantation in elderly patients with sinus node dysfunction.* Europace, 2017. **19**(7): p. 1220-1226.

8. Moola S, M.Z., Tufanaru C, Aromataris E, Sears K, Sfetcu R, Currie M, Lisy K, Qureshi R, Mattis P, Mu P., *Chapter 7: Systematic reviews of etiology and risk. In: Aromataris E, Munn Z (Editors).* JBI Manual for Evidence Synthesis. Available from <https://synthesismanual.jbi.global>. , 2020.

9. Bexton, R.S., A. Davies, and R.A. Kenny, *The rate-drop response in carotid sinus syndrome: the Newcastle experience.* Pacing Clin Electrophysiol, 1997. **20**(3 Pt 2): p. 840.

10. Karunaratne, P.M., P.A. Broadhurst, and C.A. Norris, *Outcomes of permanent pacemaker implantation for carotid sinus hypersensitivity in a district general hospital with a Falls Fits Faints and Funny Turns Clinic.* Scottish Medical Journal, 2002. **47**(6): p. 128-131.

11. Genta, F.T., et al., *Cardiac rehabilitation after transcatheter aortic valve implantation compared to patients after valve replacement.* Journal of Cardiovascular Medicine, 2017. **18**(2): p. 114-120.

12. Shea, B.J., et al., *AMSTAR 2: a critical appraisal tool for systematic reviews that include randomised or non-randomised studies of healthcare interventions, or both.* BMJ, 2017. **358**: p. j4008.

13. Gillespie, L.D., et al., *Interventions for preventing falls in older people living in the community.* Cochrane Database Syst Rev, 2012. **2012**(9): p. CD007146.

14. Dautzenberg, L., et al., *Interventions for preventing falls and fall-related fractures in community-dwelling older adults: A systematic review and network meta-analysis.* J Am Geriatr Soc, 2021. **69**(10): p. 2973-2984.

15. Perego, F., et al., *The Dilemma of Falls in Older Persons: Never Forget to Investigate the Syncope.* Medicina (Kaunas), 2021. **57**(6).

16. Romagnolo, A., et al., *Cardiovascular autonomic neuropathy and falls in Parkinson disease: a prospective cohort study.* J Neurol, 2019. **266**(1): p. 85-91.

17. de Ruiter, S.C., et al., *Multiple causes of syncope in the elderly: diagnostic outcomes of a Dutch multidisciplinary syncope pathway.* Europace, 2018. **20**(5): p. 867-872.

18. Ungar, A., et al., *Etiology of Syncope and Unexplained Falls in Elderly Adults with Dementia: Syncope and Dementia (SYD) Study.* J Am Geriatr Soc, 2016. **64**(8): p. 1567-73.

19. Wold, J.F.H., et al., *A multidisciplinary care pathway for the evaluation of falls and syncope in geriatric patients.* European Geriatric Medicine, 2015. **6**(5): p. 487-494.

20. Parry, S.W. and R.A. Kenny, *Drop attacks in older adults: systematic assessment has a high diagnostic yield.* J Am Geriatr Soc, 2005. **53**(1): p. 74-8.

21. Davies, A.J. and R.A. Kenny, *Falls presenting to the accident and emergency department: types of presentation and risk factor profile.* Age Ageing, 1996. **25**(5): p. 362-6.

22. Vilches-Moraga, A., et al., *Midodrine hydrochloride in the management of older adults with neurocardiogenic syncope and orthostatic hypotension: A prospective observational study.* European Geriatric Medicine, 2012. **3**(5): p. 295-298.

23. Hohtari-Kivimaki, U., et al., *Orthostatic Hypotension is a Risk Factor for Falls Among Older Adults: 3-Year Follow-Up.* J Am Med Dir Assoc, 2021. **22**(11): p. 2325-2330.

24. Juraschek, S.P., et al., *Comparison of supine and seated orthostatic hypotension assessments and their association with falls and orthostatic symptoms.* J Am Geriatr Soc, 2022. **70**(8): p. 2310-2319.

25. Mol, A., et al., *Orthostatic Blood Pressure Recovery Measured Using a Sphygmomanometer Is Not Associated with Physical Performance or Number of Falls in Geriatric Outpatients.* Gerontology, 2022. **68**(1): p. 75-79.

26. Welmer, A.K., et al., *Associations of blood pressure with risk of injurious falls in old age vary by functional status: A cohort study.* Exp Gerontol, 2020. **140**: p. 111038.

27. Mol, A., et al., *Orthostatic Hypotension and Falls in Older Adults: A Systematic Review and Meta-analysis.* J Am Med Dir Assoc, 2019. **20**(5): p. 589-597 e5.

28. van der Velde, N., et al., *Measuring orthostatic hypotension with the Finometer device: is a blood pressure drop of one heartbeat clinically relevant?* Blood Pressure Monitoring, 2007. **12**(3): p. 167-171.

29. Aronow, W.S. and C. Ahn, *Postprandial hypotension in 499 elderly persons in a long-term health care facility.* J Am Geriatr Soc, 1994. **42**(9): p. 930-2.

30. Aronow, W.S. and C. Ahn, *Association of postprandial hypotension with incidence of falls, syncope, coronary events, stroke, and total mortality at 29-month follow-up in 499 older nursing home residents.* J Am Geriatr Soc, 1997. **45**(9): p. 1051-3.

31. Donoghue, O.A., et al., *Is orthostatic hypotension and co-existing supine and seated hypertension associated with future falls in community-dwelling older adults? Results from The Irish Longitudinal Study on Ageing (TILDA).* PLoS One, 2021. **16**(5): p. e0252212.

32. Moloney, D., et al., *Clinical clustering of eight orthostatic haemodynamic patterns in The Irish Longitudinal Study on Ageing (TILDA).* Age Ageing, 2021. **50**(3): p. 854-860.

33. Mol, A., et al., *Blood Pressure Drop Rate After Standing Up Is Associated With Frailty and Number of Falls in Geriatric Outpatients.* J Am Heart Assoc, 2020. **9**(7): p. e014688.

34. Finucane, C., et al., *Impaired Orthostatic Blood Pressure Recovery Is Associated with Unexplained and Injurious Falls.* J Am Geriatr Soc, 2017. **65**(3): p. 474-482.

35. Goh, C.H., et al., *Standing beat-to-beat blood pressure variability is reduced among fallers in the Malaysian Elders Longitudinal Study.* Medicine (Baltimore), 2017. **96**(42): p. e8193.

36. Goh, C.H., et al., *Evaluation of Two New Indices of Blood Pressure Variability Using Postural Change in Older Fallers.* Medicine (Baltimore), 2016. **95**(19): p. e3614.

37. Jonas, M., R. Kazarski, and G. Chernin, *Ambulatory blood-pressure monitoring, antihypertensive therapy and the risk of fall injuries in elderly hypertensive patients.* Journal of Geriatric Cardiology, 2018. **15**(4): p. 284-289.

38. Le Couteur, D.G., et al., *Postprandial systolic blood pressure responses of older people in residential care: association with risk of falling.* Gerontology, 2003. **49**(4): p. 260-4.

39. Puisieux, F., et al., *Ambulatory blood pressure monitoring and postprandial hypotension in elderly persons with falls or syncopes.* J Gerontol A Biol Sci Med Sci, 2000. **55**(9): p. M535-40.

40. Turusheva, A., et al., *Association Between Arterial Stiffness, Frailty and Fall-Related Injuries in Older Adults.* Vascular Health & Risk Management, 2020. **16**: p. 307-316.

41. Wong, A.K., et al., *High arterial pulse wave velocity is a risk factor for falls in community-dwelling older people.* J Am Geriatr Soc, 2014. **62**(8): p. 1534-9.

42. Isik, M., et al., *Blunted baroreflex sensitivity: An underestimated cause of falls in the elderly?* European Geriatric Medicine, 2012. **3**(1): p. 9-13.

43. Davison, J., S. Brady, and R.A. Kenny, *24-hour ambulatory electrocardiographic monitoring is unhelpful in the investigation of older persons with recurrent falls.* Age Ageing, 2005. **34**(4): p. 382-6.

44. Rosado, J.A., et al., *The value of Holter monitoring in evaluating the elderly patient who falls.* J Am Geriatr Soc, 1989. **37**(5): p. 430-4.

45. Gordon, M., M. Huang, and C.I. Gryfe, *An evaluation of falls, syncope, and dizziness by prolonged ambulatory cardiographic monitoring in a geriatric institutional setting.* J Am Geriatr Soc, 1982. **30**(1): p. 6-12.

46. Midttun, M., *Diagnosis effectiveness of arrhythmia with R-test electrocardiogram in old fallers.* European Geriatric Medicine, 2011. **2**(5): p. 270-272.

47. Bhangu, J., et al., *Long-term cardiac monitoring in older adults with unexplained falls and syncope.* Heart, 2016. **102**(9): p. 681-686.

48. Maggi, R., et al., *Additional diagnostic value of implantable loop recorder in patients with initial diagnosis of real or apparent transient loss of consciousness of uncertain origin.* Europace, 2014. **16**(8): p. 1226-30.

49. Martinez, P., et al., *[Experience with the use of an implantable loop recorder in a series of older people with falls and suspected arrhythmic syncopes].* Rev Esp Geriatr Gerontol, 2014. **49**(3): p. 121-4.

50. Armstrong, V.L., et al., *The use of an implantable loop recorder in the investigation of unexplained syncope in older people.* Age Ageing, 2003. **32**(2): p. 185-8.

51. Jansen, S., et al., *The Association of Cardiovascular Disorders and Falls: A Systematic Review.* J Am Med Dir Assoc, 2016. **17**(3): p. 193-9.

52. Sachpekidis, V., et al., *Carotid sinus hypersensitivity is common in patients presenting with hip fracture and unexplained falls.* Pacing Clin Electrophysiol, 2009. **32**(9): p. 1184-90.

53. Kerr, S.R., et al., *Carotid sinus hypersensitivity in asymptomatic older persons: implications for diagnosis of syncope and falls.* Arch Intern Med, 2006. **166**(5): p. 515-20.

54. Richardson, D.A., et al., *How reproducible is the cardioinhibitory response to carotid sinus massage in fallers?* Europace, 2002. **4**(4): p. 361-364.

55. Schoon, Y., et al., *Head turning-induced hypotension in elderly people.* PLoS One, 2013. **8**(8): p. e72837.
